# A unified route for flavivirus structures uncovers essential pocket factors conserved across pathogenic viruses

**DOI:** 10.1038/s41467-021-22773-1

**Published:** 2021-06-01

**Authors:** Joshua M. Hardy, Natalee D. Newton, Naphak Modhiran, Connor A. P. Scott, Hariprasad Venugopal, Laura J. Vet, Paul R. Young, Roy A. Hall, Jody Hobson-Peters, Fasséli Coulibaly, Daniel Watterson

**Affiliations:** 1 Infection and Immunity Program, Biomedicine Discovery Institute and Department of Biochemistry and Molecular Biology, Monash University, Clayton, VIC Australia; 2 Australian Infectious Diseases Research Centre, School of Chemistry and Molecular Biosciences, The University of Queensland, Brisbane, QLD Australia; 3 Ramaciotti Centre for Cryo-Electron Microscopy, Monash University, Clayton, VIC Australia

**Keywords:** Dengue virus, Virus structures, Cryoelectron microscopy

## Abstract

The epidemic emergence of relatively rare and geographically isolated flaviviruses adds to the ongoing disease burden of viruses such as dengue. Structural analysis is key to understand and combat these pathogens. Here, we present a chimeric platform based on an insect-specific flavivirus for the safe and rapid structural analysis of pathogenic viruses. We use this approach to resolve the architecture of two neurotropic viruses and a structure of dengue virus at 2.5  Å, the highest resolution for an enveloped virion. These reconstructions allow improved modelling of the stem region of the envelope protein, revealing two lipid-like ligands within highly conserved pockets. We show that these sites are essential for viral growth and important for viral maturation. These findings define a hallmark of flavivirus virions and a potential target for broad-spectrum antivirals and vaccine design. We anticipate the chimeric platform to be widely applicable for investigating flavivirus biology.

## Abstract (web summary)

Understanding virus assembly could identify potential drug targets. Here the authors use a safe and efficient method to solve pathogenic flavivirus structures, revealing two lipid-like ligands within highly conserved pockets of the stem region of envelope protein that are important for virus maturation.

## Introduction

The flavivirus genus of the *Flaviviridae* family represents a large and diverse group of viruses, including several major human pathogens such as dengue virus (DENV), Zika virus (ZIKV), and Japanese encephalitis virus (JEV). Depending on the virus and the host response, symptoms of flavivirus infections range from mild fevers to haemorrhagic symptoms, microcephaly and fatal encephalitis^1^. The family has a diverse host range. Most are transmitted by arthropods including mosquitoes and ticks to vertebrate hosts. However, some viruses, known as insect-specific flaviviruses (ISFs), have no known vertebrate hosts and are restricted to replication in mosquitoes^2^. Several medically important flaviviruses are endemic, persisting in animal reservoirs such as wild birds or horses, heightening the need for effective vaccines and treatments.

With limited vaccines and no targeted therapeutics available, flaviviruses continue to pose a significant global threat to human health^3^. DENV causes an estimated 400 million cases a year^4^. Vaccine development has been complicated by the co-circulation of four serotypes and the risk of disease enhancement linked to non-neutralizing immune responses^5^. Population growth and climate change have increased the reach of flaviviruses such as West Nile virus (WNV) that have previously only caused local and regional epidemics but are now prevalent in multiple continents^6,7^. The distribution of ZIKV has expanded from a narrow tropical localization across Africa and Asia to a global presence including the Americas and South-East Asia^8^. The ability to respond rapidly to emerging flaviviruses is key to enabling therapeutic and vaccine development in a timely manner and reducing the toll of viral epidemics.

Flaviviruses are positive-strand RNA viruses with enveloped virus particles. The RNA genome is translated into a polyprotein, which is cleaved by viral and host proteases to release three structural proteins (C, prM, and E) and seven non-structural proteins (NS1, NS2A, NS2B, NS3, NS4A, NS4B, and NS5). The flavivirus virion consists of an asymmetric ribonucleocapsid core^9^ (C and RNA) surrounded by an icosahedral layer composed of the E and prM/M proteins anchored to a lipid bilayer^10^. The E protein is responsible for attachment to target cells and mediates fusion between the viral and cellular membranes during entry.

Flaviviruses undergo a complex maturation process that prevents premature activation before cell egress^11^. Virions bud into the endoplasmic reticulum as non-infectious immature particles that contain projecting spikes of prM-E trimers. During the transit of the virion through the secretory pathway, acidification and cleavage of prM into pr and M induce large rearrangements of the spike at the viral surface, ultimately resulting in antiparallel homodimers arranged in a characteristic herringbone pattern. Upon secretion of the virus, the pr component is lost resulting in a fusion-competent virus^12,13^.

Most flavivirus vaccine candidates target E to prevent entry by blocking attachment or fusion^14^. Analysis of E by X-ray crystallography has provided high-resolution structures delineating the molecular determinants of important neutralizing epitopes such as a cross-reactive E dimer epitope^15^. However, some of the antigenic and functional features of flaviviruses can only be explained in the context of the infectious particle^16–22^. These quaternary interactions are best characterized by cryo-electron microscopy (cryo-EM), but, while structures of flavivirus virions have been solved up to a resolution of 3.1 Å^23^, most reconstructions have been limited to a lower resolution by sample heterogeneity. In addition, cryo-EM analysis of the native virions of highly pathogenic or newly emerging viruses is only feasible in a very limited number of facilities that can ensure the appropriate level of containment throughout the experiment. Here, we show how chimeras between an ISF and pathogenic viruses provides a streamlined pathway to structure determination of flaviviruses.

**Fig. 1:**
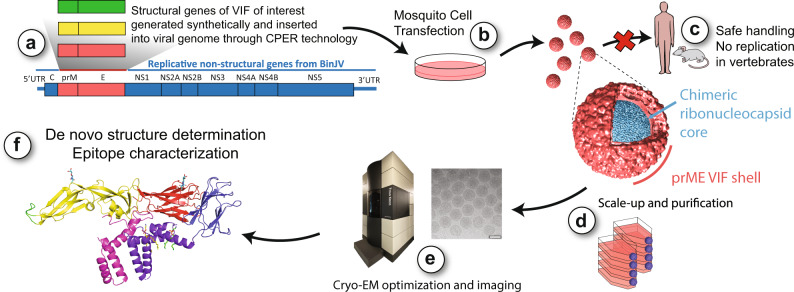
A chimeric platform for high-resolution structure determination of flaviviruses particles by cryo-EM. **a** Structural genes encoding prM and E proteins from a vertebrate-infecting flavivirus (VIF) are used to replace the homologous genes of the ISF, BinJV, via circular polymerase extension reaction (CPER) methodology. **b** CPER product is transfected into insect cells to produce a chimeric virus that encodes the replicative machinery of the ISF, but the structural components of the VIF. **c** Safety and ease-of-use is ensured as the chimeric virus cannot replicate in vertebrate cells. **d** Chimeric virus production is scaled up and virions purified for TEM analysis. **e** Cryo-EM imaging is performed on vitrified virus particles. Scale bar indicates 50 nm. **f** Single-particle analysis is used to generate high-resolution virion electron density maps, allowing de novo structure elucidation at the atomic scale to inform rational design of new vaccines, therapeutics and diagnostics.

The system uses the ISF Binjari virus (BinJV), which we have previously shown to be highly tolerant of foreign prM-E genes from pathogenic viruses while retaining restriction to mosquito cells^24^. The exact mechanisms that underpin BinJV host restriction have not been fully defined, but are multifactorial and could be expected to restrict the replication of any prM-E chimeric viruses that contain the full non-structural, untranslated and capsid elements from BinJV^25^. Chimeric particles are constructed using a streamlined amplicon-based method, which does not require intermediate plasmid vectors or prokaryotic hosts and is directly compatible with synthetic DNA fragment-based construction (Fig. 1) The chimeric particles produced cannot replicate in vertebrate hosts and elicit neutralizing responses to wild-type viruses, indicating that they authentically present protective epitopes^24,26^. Here, we used the system to investigate flavivirus structures at the atomic level by cryo-EM.

The resolutions achieved are among the best for enveloped viruses allowing accurate modelling of the surface proteins and, in favourable cases, the identification of water molecules and ligands. We use this approach to reveal the presence of two unrecognized binding sites in the stem of E occupied by lipid-like pocket factors. We show that these sites are conserved, essential for viral growth and extensively remodelled during maturation. Together, these findings identify an attractive target for the development of broad-spectrum assembly inhibitors and stabilized vaccine designs.

## Results

### Chimeric viruses recapitulate the structure of wild-type virions

**Fig. 2:**
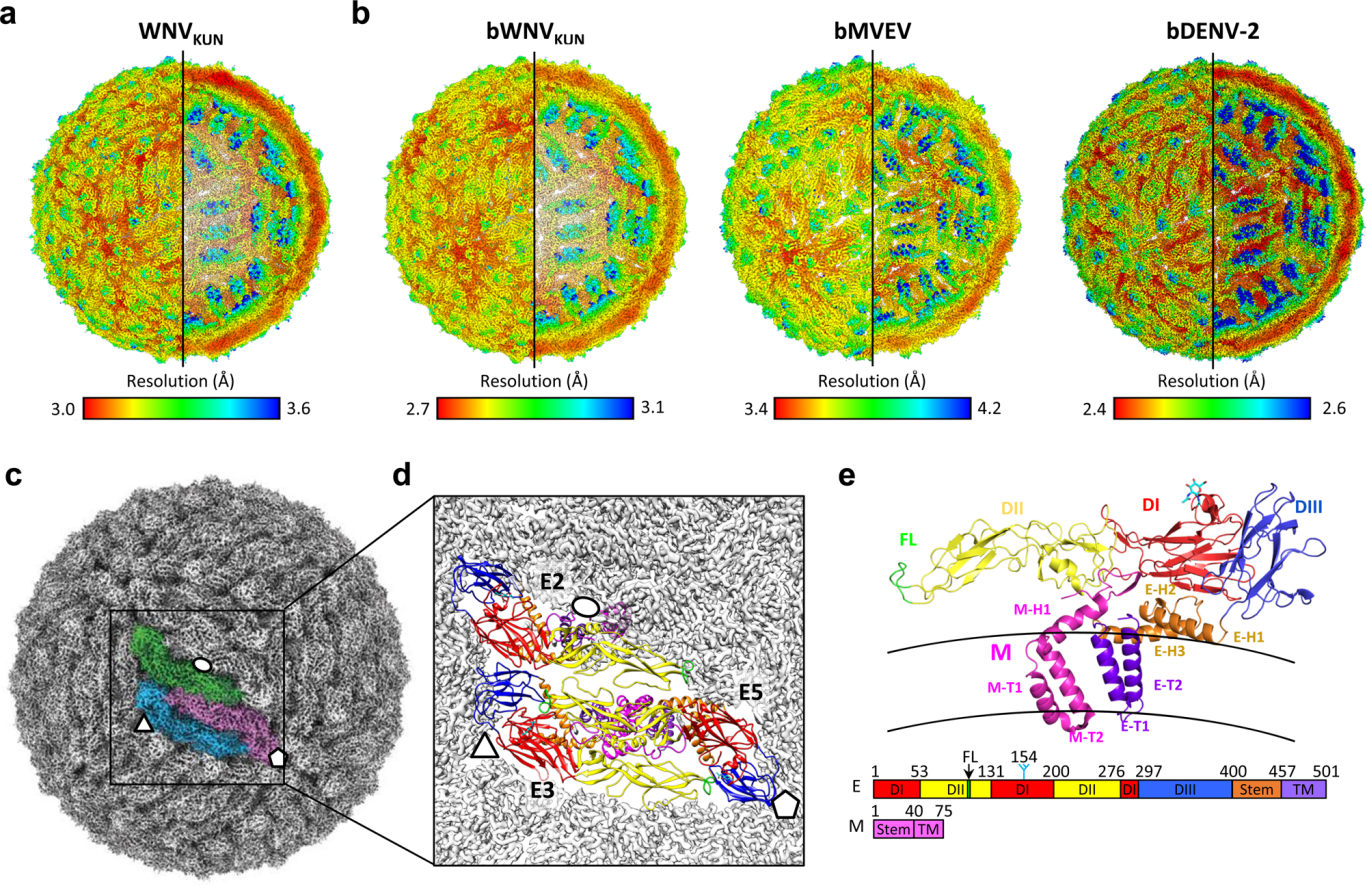
A chimeric platform facilitates high-resolution structure determination of flaviviruses particles. **a**, **b** Cryo-EM reconstructions of **a** WNV_KUN_ and **b** the chimeric viruses bWNV_KUN_, bMVEV and bDENV-2 coloured by local resolution in a rainbow gradient from high (red) to low (blue). **c** The asymmetric unit of WNV_KUN_ is composed of three copies of E (green, pink and blue) and M (not visible). Ellipses, triangles and pentagons represent the 2-, 3- and 5-fold icosahedral axes, respectively. **d** Asymmetric unit displayed as a cartoon and coloured by domain. Each E protein is labelled according to its proximity to the icosahedral symmetry axes. **e** A cartoon representation of the WNV_KUN_ structure coloured by domain according to the domain diagram below: E ectodomain 1 (DI, red) containing an N-linked glycan at Asn154 (cyan); E ectodomain 2 (DII, yellow) containing a fusion loop (FL, green); E ectodomain 3 (DIII, blue); E membrane domain (purple) consisting of a stem of three membrane-proximal helices (E-H1, E-H2 and E-H3) and two transmembrane helices (E-T1 and E-T2); M membrane domain (magenta) consisting of a stem helix (M-H1) and two transmembrane helices (M-T1 and M-T2).

**Fig. 3:**
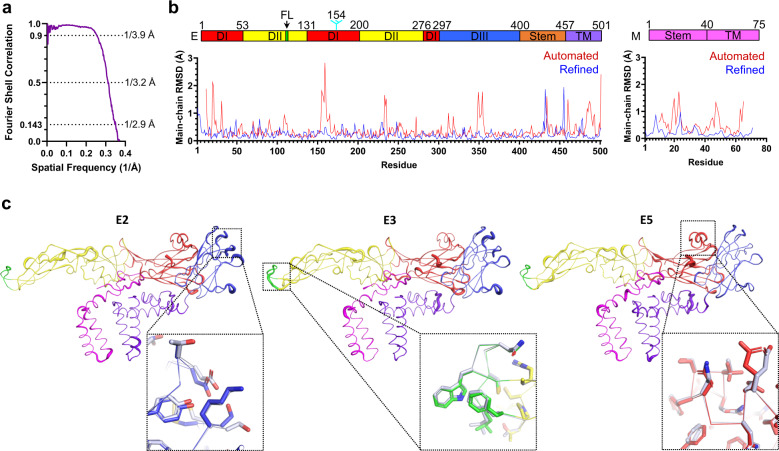
Chimeric bWNV_KUN_ recapitulates the structure of wild-type WNV_KUN_. **a** FSC of the masked maps of bWNV_KUN_ and WNV_KUN_. **b** Plot of the root-mean-squared deviation (RMSD) between pairs of main-chain atoms for equivalent residues of the automated build of bWNV_KUN_ and WNV_KUN_ (red), and the best refined models (blue). The corresponding domain organization of WNV_KUN_ is shown as in Fig. 2e. **c** A ribbon representation of the three copies of E within the asymmetric unit of bWNV_KUN_ coloured as in **b**. The ribbon thickness is proportional to the local RMSD differences between bWNV_KUN_ and WNV_KUN_ including all equivalent atoms. Differences are very small with an RMSD per residue range of 0.02–1.36 Å and an average of 0.41 Å, and primarily located in exposed loops. Selected zoom panels show a close agreement even in regions of maximal RMSD. Side chains are shown as sticks for bWNV_KUN_ (same colouring as ribbon) and WNV_KUN_ (white).

To confirm the structural mimicry of the chimeric viruses at the atomic level we determined the cryo-EM reconstructions of wild-type WNV_KUN_ (Kunjin strain—KUN), which can be handled in BSL2 equivalent cryo-EM facilities^27,28^, and the chimeric counterpart BinJV-WNV_KUN_ (bWNV_KUN_). Viruses were gradient purified, and purity was confirmed by sodium dodecyl sulfate-polyacrylamide gel electrophoresis (SDS-PAGE) (Supplementary Fig. 1). Single-particle analysis of cryo-EM images (Supplementary Table 1) produced high-resolution reconstructions for WNV_KUN_ and bWNV_KUN_ with an average resolution of 3.1 and 2.9 Å, respectively (Supplementary Fig. 2 and Fig. 2a, b). When compared by Fourier shell correlation, the two masked maps are nearly identical up to a resolution of 3.9 Å with a correlation coefficient >90% (50% at 3.2 Å) (Fig. 3a). The electron density is well defined for the M and E proteins. As for most flavivirus structures, the internal capsid does not follow the icosahedral symmetry and is not interpretable in our reconstructions.

The asymmetric unit of WNV_KUN_ consists of three copies of E and M with a herringbone arrangement of antiparallel E dimers typical of flaviviruses (Fig. 2c). E is composed of three canonical ectodomains: a central β-barrel domain (DI); an elongated dimerization domain (DII) containing a fusion peptide (residues 98–110); and an Ig-like domain (DIII), which connects to the membrane domain in the lipid bilayer^29^ (Fig. 2d). The C-terminal domain forms a stem consisting of three membrane-proximal helices (E-H1, E-H2 and E-H3) and two transmembrane helices (E-T1 and E-T2) (Fig. 2e). M is located underneath the E dimer on the membrane side. It consists simply of a membrane-proximal helix (M-H1) and two transmembrane helices (M-T1 and M-T2) (Fig. 2e).

To provide an unbiased comparison between the native and chimeric structures, we performed automated model building for both maps using a relatively distant template (ZIKV prM-E PDB:6CO8, 53% sequence identity to WNV_KUN_) showing that the two virions are remarkably similar (Fig. 3b). Alignment of the six molecules in the asymmetric unit as a block (3 × M, 3 × E) showed a very close match between the models with an overall root-mean-squared deviation (RMSD) of 0.681 Å for all atoms of 1719 equivalent residues in the final refined structures. The three independent M/E complexes show a comparable degree of similarity, with only a few conformational changes at the side chain level (Fig. 3c). The lower resolution WNV_KUN_ reconstruction has two flipped glycine residues, Gly432 and Gly455 (Supplementary Fig. 2f). This difference is likely to represent an incorrect modelling choice in ambiguous electron density. Crystal structures refined at a resolution of 3–3.1 Å are available for the ectodomain of the E protein of WNV^30–32^ providing an orthogonal validation. Direct comparison with these structures is possible once inter-domain hinge movements have been accounted for. Excluding the hinge regions, the structure of DI–III is almost identical to the crystal structure of WNV (PDB: 2I69) with an all-atom RMSD of 1.25 Å for 395 residues. We conclude that bWNV_KUN_ has a native-like structure supporting detailed analysis of functional and antigenic features.

### Comparative structural biology of neurovirulent flaviviruses

**Fig. 4:**
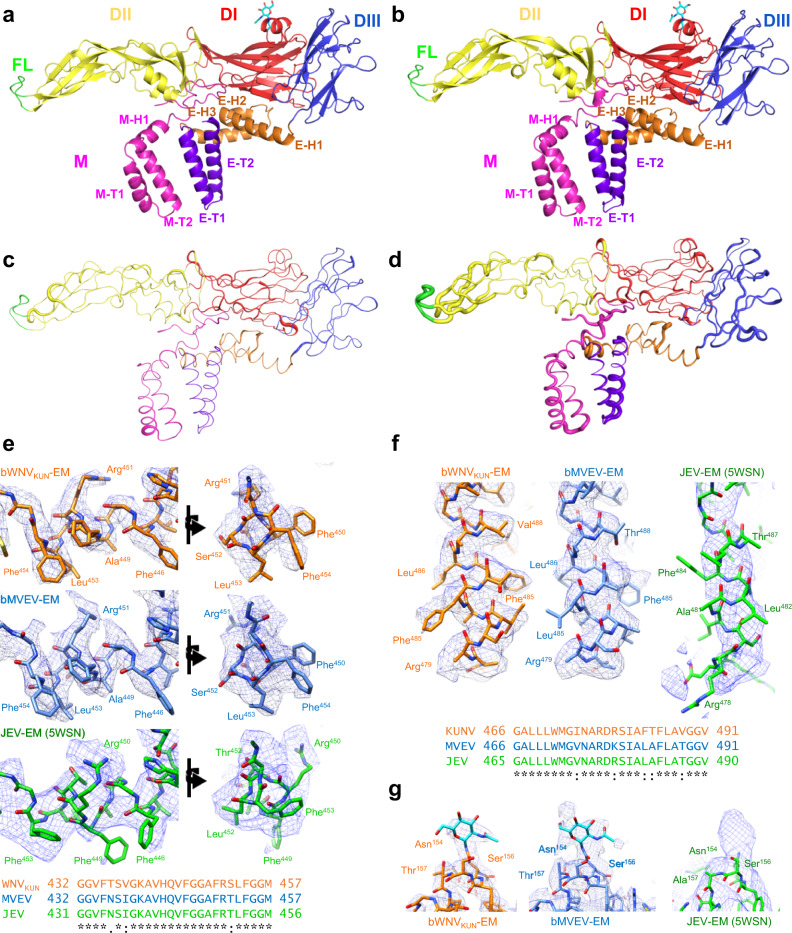
A high-resolution view of the stem and membrane regions of neurotropic flaviviruses. **a**, **b** A cartoon representation of the **a** bWNV_KUN_ and **b** bMVEV structure coloured by domain: E ectodomain 1 (DI, red); E ectodomain 2 (DII, yellow) containing a fusion loop (FL, green) and an N-linked glycan at Asn67 (cyan); E ectodomain 3 (DIII, blue); E membrane domain (purple) consisting of an ‘anchor’ of three perimembrane helices (E-H1, E-H2 and E-H3) and a ‘stem’ of two transmembrane helices (E-T1 and Ea-T2); M membrane domain (magenta) consisting of a perimembrane helix (M-H1) and two transmembrane helices (M-T1 and M-T2). **c**, **d** A ribbon representation of local RMSD differences between the structures of **c** bMVEV and bWNV_KUN_ and **d** bMVEV and JEV (PDB: 5WSN) where the ribbon diameter is proportional to the RMSD. For bMVEV/bWNV_KUN_, the RMSD ranges between 0.05 and 2.93 Å with an average of 0.64 Å, whereas bMVEV/JEV has a range of 0.09–4.81 Å and an average of 1.28 Å. **e**, **f** A comparison of the density (mesh) for **e** M-H3 and **f** E-T2 with the structure of bWNV_KUN_ (orange sticks), bMVEV (blue sticks), and a cryo-EM structure of JEV (green sticks, PDB: 5WSN, EMD-6685). A sequence alignment is displayed below and coloured accordingly. **g** The density and model of the glycosylation site at Asn^154^ for bWNV_KUN_ (orange sticks), bMVEV (blue sticks) and JEV (green sticks).

To demonstrate utility for resolving pathogenic flaviviruses, the structure of chimeric Murray Valley encephalitis virus (bMVEV) was determined at a resolution of 3.7 Å from a single dataset (Fig. 2a). Before cryo-EM analysis, the host restriction of bMVEV was assessed and confirmed to be similar to BinJV (Supplementary Fig. 1b). MVEV is a member of the JEV serocomplex of flaviviruses, which includes Capipacoré virus, JEV, Koutango virus, St. Louis encephalitis virus, Usutu virus, WNV and Yaoundé virus^33^. MVEV has the highest fatality rate of mosquito-borne viruses endemic to Australasia^34^ and is thus too pathogenic to be imaged without inactivation at most cryo-EM facilities. Like WNV_KUN_, bMVEV has the typical structure of a flavivirus^29^, where E folds into an ectodomain, a stem region, and two C-terminal transmembrane helices (Fig. 4a–c).

Significant modelling differences exist in the stem region of E (Fig. 4d, e) and the transmembrane domains of M and E (Fig. 4d, f) between bMVEV and JEV. Given the high sequence similarity and consistency between the MVEV and WNV_KUN_ structures, these differences are likely to result from ambiguous electron density at the lower resolution of 4.3 Å for the JEV structure (Fig. 4e, f). On the side of E that faces the outside of the particle, the differences between the bMVEV, WNV_KUN_ and JEV are minimal. The only notable conformational changes are located in the fusion loop region (Fig. 4d).

**Fig. 5:**
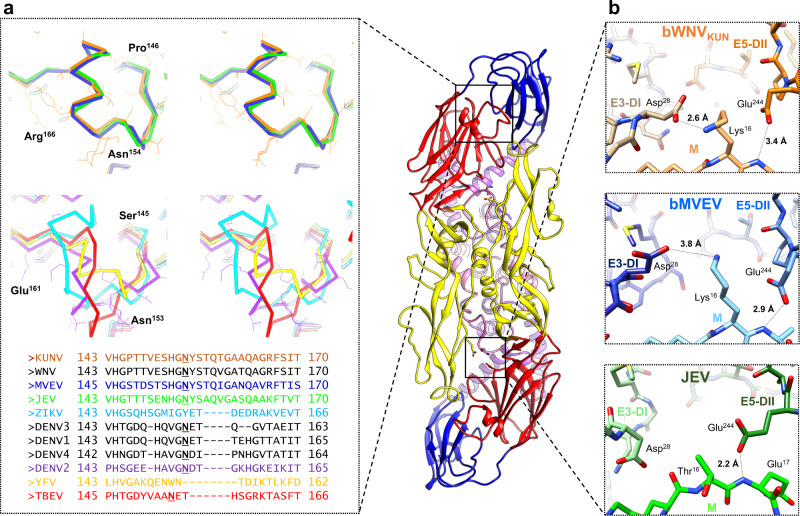
Structures of WNV_KUN_ and bMVEV highlight features involved in neurovirulence and immunogenicity. A dimer of E–M heterodimers coloured according to the domain: DI, red; DII, yellow; DIII, blue; membrane domain, purple: and M, pink. Boxes highlight the regions of interest. **a** Comparison of the backbone and sequence alignment of flavivirus structures near the Asn154 site. Structures are coloured in accordance with the sequence alignment and displayed as a wall-eye stereo view. **b** Comparison of the dimer interface between WNV_KUN_ (orange, top), bMVEV (blue, middle) and JEV (green, bottom, PDB: 5WSN). Chains are labelled with the protein name and domain. Distances are shown for hydrogen bonds.

Comparative analysis of bMVEV, bWNV_KUN_ and JEV shows that most motifs specific to encephalic viruses are conserved^35^. Residue Glu138 has been implicated in receptor binding for JEV^35^. It is in the same environment in the three structures suggesting a similar role for these viruses. As with most other flaviviruses, E is glycosylated at position Asn154 in DI (Fig. 4g). The glycosylation site at Asn154 is the immunodominant epitope in horses infected with WNV or MVEV^36^ and has been identified to be a determinant of neurovirulence for WNV_KUN_ isolates^37,38^. The Asn154 glycan site has been implicated in increased viral titres, egress from the host cell, stability at low pH and concealment of immunogenic epitopes^37,39–41^. JEV subgroup viruses contain a 4-residue insertion just after the glycan site and we find that the short helix–loop–helix structure is retained across the subgroup at this site (Fig. 5a). This structure may impact on glycan accessibility and alter possible interaction with cell surface lectins either directly, or by affecting glycan processing^42^.

By contrast, differences are observed around the hole at the centre of the E dimer for residues associated with neurovirulence in JEV. Residue Glu244 has been associated with neurovirulence^43^. In the three available structures of neurovirulent flaviviruses (Fig. 5b), Glu244 of an E subunit forms a stabilizing interaction with the main chain of residue 17 of a neighbouring M subunit. In bMVEV and bWNV_KUN_, Lys16 from M forms a salt bridge with Asp28 from the opposing E subunit (Fig. 5b), thus stabilizing the dimer formation. JEV strains vary in this region, the P3 strain previously resolved by cryo-EM retains Glu244^35^, but lacks Lys16. Attenuated strains of JEV contain a Gly at position 244, and virulence can be restored with the introduction of Glu/Asp at this site, but has only been demonstrated in the context of a Lys at position 16 in M^43^ (Fig. 5b). Mutation of Glu244 in WNV or MVEV may, therefore, provide attenuation through a shared mechanism.

**Fig. 6:**
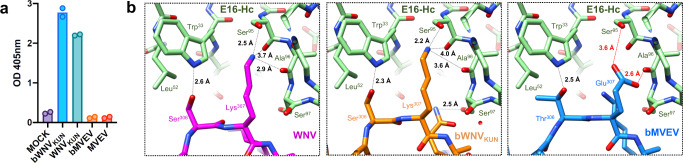
Recognition of WNV_KUN_ and bMVEV by monoclonal antibody E16. **a** E16 binding is virus-specific with no recognition of MVEV- or bMVEV-infected cells observed at 1 µg/mL. The bar graph represents the average OD from two technical replicates. **b** Structure of the WNV complex with E16 (left, PDB: 1ZTX) is compared to models of the bWNV_KUN_-E16 (middle) and bMVEV-E16 complex (right). The structures of bWNV_KUN_ and bMVEV were aligned to WNV without minimization of either component. Red lines indicate clashes.

Conformational changes are also observed at the exposed loops of DIII that have been implicated in flavivirus receptor binding^44,45^. This region is also a determinant of protective antibodies, including the E16 monoclonal antibody (mAb), which has been progressed to clinical trials for WNV treatment^46^. We show that E16 does not recognize MVEV or bMVEV, which is consistent with a Lys307Glu mutation at the centre of the epitope (Fig. 6).

### A high-resolution structure of bDENV-2

**Fig. 7:**
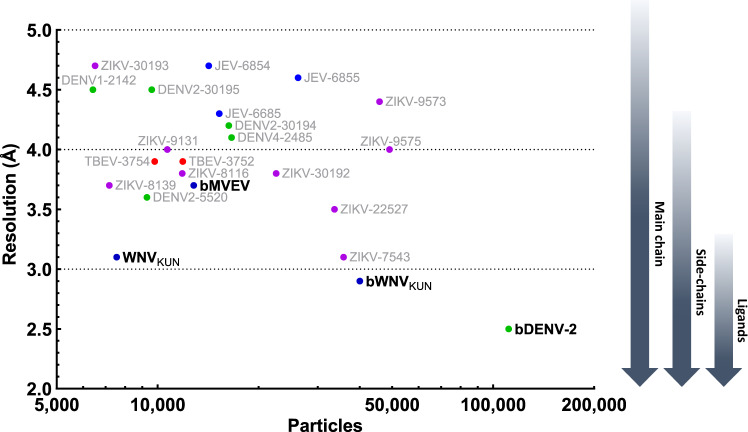
Flavivirus reconstructions using cryo-EM. Cryo-EM reconstructions of flaviviruses with a resolution higher than 5 Å are plotted against reported particle numbers on a logarithmic axis. Electron microscopy database IDs are indicated in grey next to each reconstruction. Structures are grouped as follows: DENV (green), ZIKV (magenta), JEV group (blue) and TBEV (red). The resolution range required to visualize structural details is illustrated on the right-hand side of the plot.

**Fig. 8:**
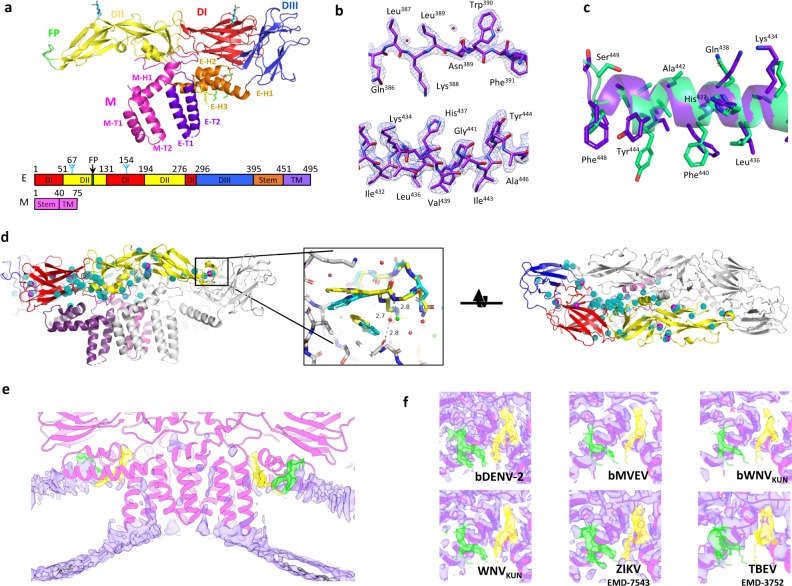
Modelling high-resolution features in bDENV-2. **a** A cartoon representation of the bDENV-2 structure coloured by domain according to the domain diagram below. The N-linked glycans are shown in cyan. **b** Zooms of the density (mesh) and fit of the bDENV-2 structure (purple sticks). Water molecules are shown as red spheres. **c** Overlay of E-H3 of bDENV-2 (purple) and DENV-2 (green, PDB: 3J27). Side chains are represented as sticks and the main chain as a cartoon. **d** Orthogonal views of the E dimer. Out of ~150 water molecules, 59 are conserved in all prM-E heterodimers within the particle and are represented as cyan dotted spheres. When compared to a crystal structure of similar resolution (PDB: 3C5X), 11 are conserved and are represented as solid, magenta spheres. The inset represents a zoom on the fusion loop highlighting a conserved water molecule. Water molecules are shown as red and green spheres for the cryo-EM structure and crystal structure, respectively. **e** A cartoon representation of an E–M dimer in bDENV-2 (magenta) subtracted from the reconstruction to produce a difference map (purple). The difference map was low-pass filtered for clarity and the density surrounding the two lipid molecules is highlighted (yellow and green). **f** A comparison of the density near the pocket factors in bDENV-2, bMVEV, bWNV_KUN_, WNV_KUN_, ZIKV (EMD-7543, PDB: 6CO8) and TBEV (EMD-3752, PDB: 5O6A). The structure of the E protein and surrounding density is displayed in magenta. The structure of the lipids from bDENV-2 is displayed in each panel as sticks (green and yellow) and density within 2 Å is coloured accordingly.

Our best reconstruction based on the BinJV platform has been determined for bDENV-2 to a resolution of 2.5 Å (Fig. 2a, and Supplementary Movie 1). This resolution jump compared to our other structures and publicly available ones was made possible, at least in part, by the high number of particles used in the reconstruction (Fig. 7). This is largely owed to the high replicative efficiency of the chimeric virus and the introduction of the WNV_KUN_ furin site, which increased homogeneity compared to wild-type DENV-2 (Supplementary Figs. 1 and 3). bDENV-2 has a similar organization as the encephalitic flaviviruses described above with the additional presence of a second glycan at positions Asn67 in DI (Fig. 8a). Density is present for ~150 water molecules for each M–E heterodimer (Fig. 8b). To our knowledge, no other cryo-EM flavivirus structure has visualized ordered water molecules in the virion. Conserved water molecules compared to crystal structures are located at structurally and functionally important interfaces including the DI/DIII interface, DII hinge region and the fusion loop (Fig. 8d). Comparison of the bDENV-2 E structure with high-resolution crystal structures of the isolated ectodomain of E is possible at the domain level. These structures are not directly comparable given that the bDENV-2 E protein is involved in multiple interactions with other E proteins, M and the underlying membrane, while the crystal structures are affected by truncation, crystal contacts and the chemical conditions of crystallization. Despite these very different environments, a structural alignment demonstrates a close agreement for 363 out of 395 residues (RMSD of 1.9 Å for all atoms—Supplementary Figure 4).

When compared to the best available cryo-EM structure of a DENV virion^47^ (3.6 Å, EMD-5520, PDB: 3J27), improvement in map quality is particularly noticeable in the membrane-proximal regions, which allows reliable modelling of the stem and TM regions of the M–E heterodimer (Fig. 8c).

### Conserved pocket factors bind the stem of E in vertebrate-infecting flaviviruses (VIFs)

**Fig. 9:**
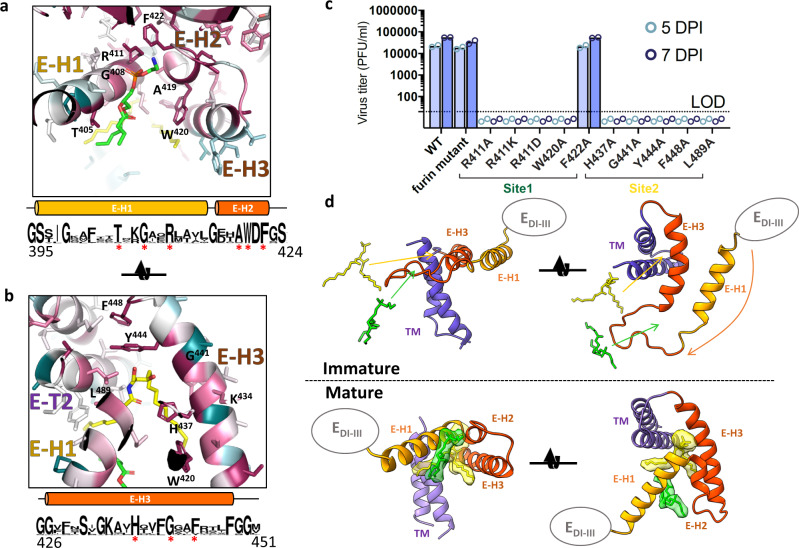
A conserved binding site in flaviviruses for lipid pocket factors. **a**, **b** Zooms of the lipid-binding sites in bDENV-2. The E protein (cartoon/sticks) is coloured by conservation from high (magenta) to low (cyan) based on an alignment of mammalian flaviviruses (Supplementary Table 4). The sequence conservation is displayed as sequence logos where amino acids at each position are listed as stacked single letter codes in a font size proportional to the frequency of occurrence. The stem helices are shown as cylinders and contact residues between E and the lipids highlighted by red asterisks. **c** Replication of viruses containing site-specific mutants was analysed at passage 1 by immunoplaque assay, recovery was only observed for mutant F422A. The open circles represent individual data point and bar graphs represent the mean value from two independent viral titrations. **d** Comparison of the TM, stem and ectodomain (E_DI–III_) of E before/after maturation across the viral membrane (left) and from the outside of the particle (right). The membrane domain is display in a cartoon with H1 (yellow), H2–H3 (orange) and the transmembrane helices (purple) labelled accordingly. For the mature structure, the lipid molecules are represented as sticks within a surface representation of the surrounding density from the reconstruction of mature bDENV-2. For the immature structure, the sequence of DENV-2 was mapped onto a previously deposited c-alpha model (PDB: 4B03) in Coot and used to generate a cartoon representation. Arrows indicate the positions of the pocket factors in the mature form. They do not represent the actual movement of lipids as their location in the immature state is unknown.

Improved modelling in the stem region revealed discrete electron densities that were not accounted for in our model (Fig. 8e). These densities have approximately the same electron density as the glycans and adjacent protein side chains, and similar shapes and volumes for the three independent subunits in the virion. Re-examination of the WNV_KUN_, bWNV_KUN_ and bMVEV maps, as well as deposited reconstructions of TBEV and ZIKV revealed unaccounted densities of similar shapes and volumes to the bDENV-2 map (Fig. 8f). Similar features were reported in two recent studies in a 3.4 Å structure of ZIKV^48^ and a 2.6 Å structure of Spondweni virus^49^. The bifurcated envelopes can accommodate a phosphatidylethanolamine molecule (site 1) and a phosphoceramide molecule (site 2), respectively, with the fatty acid tails pointing towards the membrane (Fig. 9a, b). However, the quality of the density does not allow unambiguous identification of the ligand (Supplementary Figure 5).

The two sites are located on either side of E-H1 in the stem of E. Site 1 is closer to the membrane and is largely open with sufficient space for another lipid between E-H1 and E-H3 (Fig. 9a) as suggested at low-density thresholds. Site 2 consists of a narrow gap between E-H1, E-H3 and the top of the C-terminal transmembrane helix E-T2, and a wider cavity accommodating the head group of the putative phosphoceramide molecule located underneath the ectodomain of E and near the stem of M (Fig. 9b). Both pocket factors appear to be slightly pulled out of the outer leaflet of the viral membrane through interaction with E.

Mapping of sequence conservation among VIFs onto the molecular surface of E showed that contact residues are highly conserved across the two binding sites (Fig. 9a, b). Using site-directed mutagenesis, we show that most contact residues are essential for the formation of viable virus (Fig. 9c). Specifically, residues Arg411 in E-H1 and Trp420 in E-H2 are essential in site 1 and residues His437, Gly441, Tyr444, Phe448 in E-H3 and Leu489 in E-T2 in site 2. The virus could only be recovered for mutation Phe422Ala, suggesting that this residue is dispensable to the recruitment of the site 1 ligand. However, sequencing indicated a mixed population, which is indicative that compensatory mutations may be needed to recover a viable fitness.

**Fig. 10:**
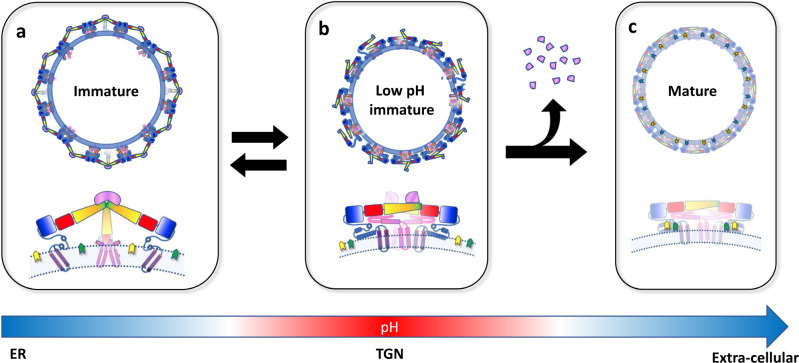
Model of the lipid ligands' role in the flavivirus virion. Cartoon representations of the virion in its **a** immature, **b** low-pH immature and **c** mature forms. The colour scheme is the same as Fig. 3 with the pr domain shown in pink like the rest of prM. The membrane is shown in blue. A blue–white–red gradient indicates acidification as the particle traffics through the secretory pathway from the endoplasmic reticulum (ER) to the trans-Golgi network (TGN) and a return to neutral pH upon secretion. The low-pH immature particle is shown with an irregular surface since it is able to breathe and potentially revert to the immature particle if acidification is reversed. Once pr is cleaved and pocket factors are acquired, the particle is irreversibly converted to the mature form.

To investigate whether the lipid-binding pockets are present in immature virions, we compared our structure with the model derived from the 6 Å cryo-EM reconstruction of immature DENV-1^50^. The ectodomains, E-H1 and TMs, are largely unchanged between the mature and immature virion apart from small inter-domain hinge movements. By contrast, the E-H2/E-H3 stem region is significantly remodelled providing a pivot point for the large-scale rearrangements associated with maturation. Compared to the unit formed by the ectodomain and E-H1 block, E-H3 is rotated by ~180° during maturation. As a result, the E-H1 surface that forms part of site 1 in the mature form is buried and inaccessible in the immature virion (Fig. 9d). Compared to the TMs, E-H3 also rotates by ~45° during maturation opening up site 2. Thus, in the immature virions, both sites are absent indicating that the pocket factors are not acquired during biogenesis of the glycoproteins and early steps of assembly. Rather, we hypothesize that they are recruited late in maturation and may play a role in the stability of the mature assembly (Fig. 10).

## Discussion

Despite, or perhaps owing to, tremendous advances in understanding flavivirus assembly, antigenicity and entry, the flow of questions on flavivirus biology that requires structural analysis is greater than ever. The chimeric platform represents a safe and robust method for investigating flavivirus structure and function. The improved safety and speed of structure determination is a methodological aspect of critical importance in the context of these viruses that have the potential to rapidly emerge and disseminate globally. Crystal structures helped determine the basis of neurovirulence in the emergence of WNV^30–32^. More recently, cryo-EM provided a model of ZIKV within less than a year of the Brazil outbreak in 2015^51,52^. Combining safety and resolution equivalent to X-ray crystallography approaches while maintaining a native-like environment of the surface proteins is particularly suited for structural analysis of these highly pathogenic flaviviruses that also present a complex antigenic landscape.

The ligand binding sites 1 and 2 are conserved in sequence and structure across VIFs defining a structural hallmark of flavivirus architecture. The binding sites are located in the stem region, which is not only important for the stability of the mature particle but also critical for both the maturation^50^ and fusion^53^ processes. A possible role for the pocket factors in stabilizing the mature virion is described in Fig. 10. We propose that the lipids are already present in the immature virion and recruited to stabilize the mature particle in the late phases of egress (Fig. 10a). The low-pH immature structure resembles the mature form but an increase in pH restores a spikey morphology (Fig. 10b). Maturation becomes irreversible upon prM cleavage and subsequent release of the pr domain (Fig. 10c). Uptake of the lipid(s) could coincide with this step, thereby compensating for the loss of pr in constraining E movements.

It will be most interesting to investigate whether small molecules are able to access sites 1 and 2 to displace the pocket factors in the assembled virion. Similar approaches have led to the development of antivirals against picornaviruses^54,55^ and HIV^56^ which destablize the virion or lock the particle in a mature form to prevent disassembly and fusion. Furthermore, our models suggest that pocket factor recruitment occurs during late-stage maturation and therefore may only require early endosomal delivery of competitive inhibitors, offering an advantage over antivirals that require uptake to the sites of replication.

The recent identification of a pocket factor within a similar membrane-proximal helical environment in the alphavirus Sindbis suggests a shared strategy for virion stabilization amongst enveloped icosahedral viruses^57^. Interestingly, the alphavirus pocket factor is also missing at low-pH^58^. It will be instructive to elucidate the role of histidine residues in these regions and the timing of the pocket factor acquisition given that alphaviruses assemble at the cell surface, while flaviviruses bud into the endoplasmic reticulum. Understanding the role of pocket factors in viral maturation and stabilization of the M and E dimer form is also likely to provide insights for vaccine design, where the presentation of highly neutralizing, maturation-dependent quaternary epitopes is a current focus for multiple flaviviruses^59,60^.

## Methods

### Cell culture

C6/36 cells (*Aedes albopictus*, RNAi-deficient; ATCC CRL1660) were maintained in Rosewell Park Memorial Institute 1640 (RPMI) supplemented with 5% foetal bovine serum (FBS) at 28 °C. BSR (*Mesocricetus auratus*, baby hamster kidney) and Vero (*Cercopithecus aethiops*, African green monkey kidney) were maintained at 37 °C 5% CO_2_ in Dulbecco’s modified Eagle’s medium (DMEM) containing 5% FBS. All media contained 50 µg/mL streptomycin, 50 U/mL penicillin and 2 mM l-glutamine.

### Virus strains and stock preparation

The viruses used in this study include BinJV (MG587038)^24^, WNV_KUN_ (JN887352)^27^, MVEV-51 (AF161266) and DENV-2-ET200 (EF440433). Virus stocks were propagated on C6/36 cell monolayers at a multiplicity of infection (MOI) of 0.1 and harvested 5–7 days post infection (d.p.i.). Viruses were clarified at 3000 × g, 4 °C for 30 min before supplementing with additional FBS for a final concentration of 10% and stored at −80 °C. Virus titres were determined by 50% tissue culture infective dose (TCID_50_)^61^ using enzyme-linked immunoabsorbant assay (ELISA). Approximately 1 × 10^4^ cells/well of C6/36 cells were seeded in a 96-well plate 5% FBS RPMI media, 24 h prior to infection. The virus was serially diluted 10-fold with 50 µL of each dilution added to ten replicate wells. The media were removed 5 d.p.i. and the cells were fixed with fixative buffer (20% acetone and 0.02% bovine serum albumin in PBS) for 2 h at 4 °C and then air-dried. The cells were blocked with blocking buffer [0.05 M Tris-HCl (pH 8.0), 1 mM EDTA, 0.15 M NaCl, 0.05% (v/v) Tween-20, and 0.2% (w/v) casein], for 1 h. The cells were probed with a pan-flavivirus anti-NS1 antibody, 4G4^62^ (1:100 dilution of hybridoma supernatant), for 1 h at 37 °C before washing in triplicate with PBS/T (0.05% Tween in PBS). Horseradish peroxidase-conjugated goat anti-mouse antibody (Dako) was added (50 µL/well) at 1:3000 dilution for 1 h at 37 °C. The cells were washed in triplicate and substrate solution (1 mM 2,2-azino- bis(3-ethylbenzthiazoline-6-sulfonic acid) and 3 mM H_2_O_2_ in a buffer prepared by mixing 0.1 M citric acid with 0.2 M Na_2_HPO_4_ to give a pH of 4.2) was added (100 µL) for 1 h in the dark before reading the OD at 405 nm.

### Immunoplaque assay (IPA)

Ten thousand cells per well of C6/36 cells were seeded in a 96-well plate overnight. Virus samples were diluted in 10-fold serial dilutions in RPMI supplemented with 2% FBS and 50 μL from each dilution was added into each well containing the cells, and incubated at 28 °C. Two-hour post infection, 150 μL of overlay media (2× M199 medium, 2% FBS, 50 U/mL penicillin, 50 µg/mL streptomycin, 2% carboxymethyl cellulose) was added. Seventy-two hours after infection, the overlay was removed and 100 μL of ice-cold 80% acetone in PBS was added and incubated at −20 °C for 30 min to fix the cells, and then air-dried. The plate was first blocked for 1 h at 37 °C with 100 µL of blocking solution (KPL, SeraCare), followed by the addition of primary 50 µL of mAb 4G4 for 1 h at 37 °C. Plates were then washed three times with PBS/T. Secondary antibody (IRDye 800CW Goat anti-human, LI-COR, USA) at 1:2500 dilution was added (50 µL/well) and incubated for 1 h at 37 °C in the dark. Plates were washed three times, air-dried and then kept in the dark at room temperature until ready for imaging. Plates were scanned using the LI-COR Biosciences Odyssey Infrared Imaging System. Viral titres from IPA are expressed as focus-forming units per mL. The results were plotted in GraphPad Prism (v. 8.1.2, La Jolla, CA, USA).

### Generation of chimeric virus particles and mutagenesis

The bVIF chimeric particles were generated using the circular polymerase extension reaction (CPER) as previously described^24^. RNA of BinJV, WNV_KUN_, MVEV and DENV-2 was extracted (NucleoSpin RNA) and converted to complementary DNA (Superscript III Reverse Transcriptase; Invitrogen). The prM and E genes of each VIF were amplified along with the five BinJV-derived fragments, which all contained complementary overhangs with the subsequent gene^24^. Primers are provided in Supplementary Table 2. The linker region connecting the 5′- and 3′-untranslated region contained a modified OpiE2 promoter (Supplementary Note 1) as previously described^63^. To optimize prM cleavage in DENV-2 prM-E, positions 1–7 of the DENV-2 furin cleavage site were exchanged with those of WNV_KUN_ using two overlapping mutagenesis PCR products (Supplementary Fig. 3). Each fragment was purified and added in equimolar proportions (0.1 pmol) to a Q5 PCR reaction (NEB) and incubated as per the following: 98 °C 3 min; 2× [98 °C 30 s, 55 °C 30 s, 72 °C 6 min]; 10× [98 °C 30 s, 55 °C 30 s, 72 °C 8 min]. The reaction was transfected into C6/36 cells using Effectene (Qiagen) as per the manufacturer’s guidelines. Successful recovery of chimeric virus was tested by immunofluorescence assay. Virus titres were determined by TCID_50_ fixed cell ELISA or IPA. The binding profile of E16 with WNV_KUN_, bWNV_KUN_, MVEV and bMVEV was assessed by fixed-cell ELISA outlined above. C6/36 cells were infected at an MOI of 0.1, fixed 5 d.p.i. and probed with recombinant E16 antibody^46^ at 1 μg/ml.

Point mutation R441A, R441D, R441K, W420A, F422A, H437A, G441A, Y444A, F448A and L489A were introduced onto a prM and E PCR product via two overlapping mutagenesis PCR, using a combination of forward and reverse mutagenesis primers (Supplementary Table 2). The amplicon containing complementary overhangs was incorporated into the BinJV genome backbone, which was transfected into C6/36 cells to produce passage 0 (P0) virus stock. The P0 stocks were then titered by IPA and used for further experiments experiment.

### Immunofluorescence assay

Infected cell monolayers prepared on glass coverslips were fixed with 100% ice-cold acetone for 1–2 min, dried and stored at −20 °C until required. Coverslips were blocked for 1 h before probing with a 1:10 dilution of pan-flavi anti-NS1 antibody 4G4^62^ for 1 h. Fixed cells were washed three times with PBS/T and 1:1000 dilution of AlexaFluor 488-conjugated goat anti-mouse IgG (H + L) (Invitrogen) for 30 min. The cell nuclei were stained with 1:1000 dilution of Hoeschst 33342 (Thermo Scientific) for 5 min before washing three times with PBST. All steps were performed at room temperature with rocking and diluted in TENTC blocking buffer. Coverslips were mounted onto glass slides using ProLong Gold antifade mountant (Thermo Scientific) and imaged using an LSM510 confocal microscope.

### In vitro host range assessment

To assess the ability for viruses to infect and replicate in vertebrate cells, C6/36 cells and selected vertebrate cells were seeded onto glass coverslips at 1 × 10^5^ per mL 24 h before infection. Cells were then infected with bMVEV, BinJV, WNV_KUN_ or mock-infected at an MOI of 1 and incubated for 1 h at 37 °C. The coverslips were washed three times with sterile PBS before replacing the media and incubating for 7 days. The virus supernatant was stored at −80 °C. The coverslips were fixed with 100% acetone and IFA was conducted.

### Purification of flavivirus particles

Each virus was grown in C6/36 cells with RPMI-1640 medium supplemented with 2% FBS. bWNV_KUN_, WNV_KUN_ and bMVEV were inoculated onto C6/36 cells at an MOI of 0.1. bWNV_KUN_ and WNV_KUN_ were harvested 2, 6 and 10 d.p.i. with the cells replenished with media after each harvest. bMVEV was harvested 3, 6 and 9 d.p.i. bDENV-2 was infected at an MOI of 0.01 and harvested 3, 5 and 7 d.p.i. Virus supernatant was clarified at 3000 × *g* for 30 min at 4 °C after every harvest. The virus was precipitated using 8% polyethylene glycol (PEG8000) overnight before centrifugation for 1 h at 4 °C, 8000 × g using an Avanti J-26 JLA10.5 rotor. The virus pellet was resuspended in NTE (12 mM Tris at pH 8, 120 mM NaCl, 1 mM EDTA pH 8) buffer prior to ultracentrifugation (133,907 × *g*, 2 h, 4 °C; Beckman Coulter SW32) through a 20% sucrose cushion. The precipitated virus pellet was resuspended in NTE and then clarified at 5000 × *g* for 10 min with the resulting supernatant gradient purified using a 25–40% potassium tartrate gradient (336,840 × *g*, 1 h, 4 °C; Beckman Coulter SW60). The virus bands resolved by the gradient were harvested and buffer exchanged into NTE and stored at 4 °C. The purity and concentration were determined by unreduced SDS-PAGE followed by SYPRO™ Ruby protein stain (Invitrogen).

### Cryo-electron microscopy

An aliquot of 3.5 µL of purified viral particles was applied to a glow-discharged R1.2/1.3 holey carbon grid (Quantifoil Micro Tools GmbH, Germany). The grid was blotted and plunge-frozen in liquid ethane using a Vitrobot Mark IV (FEI/Thermo Fisher Scientific) (Supplementary Table 3). Grids were transferred under liquid nitrogen to a Titan Krios transmission EM (FEI/Thermo Fisher Scientific) operated at 300 kV and set for parallel illumination. Movies were recorded using EPU 2 (FEI) on a K2 Summit direct electron detector (Gatan Inc., USA) in super-resolution mode with energy filtering. The data collection parameters varied for each sample and are summarized in Supplementary Table 1.

### Image processing and single-particle reconstruction

The movies were binned two times by Fourier cropping before motion correction and integrated with MotionCor2 (v. 1.1.0)^64^, giving a final pixel size of 1.04/1.34 Å. The contrast transfer function (CTF) parameters of each image were determined using Gctf (v. 1.06)^65^ and images with significant astigmatism or drift were removed. The remaining micrographs were used for particle picking and 3D reconstruction.

Using RELION (v. 3.1)^66^, particles were manually selected from a subset of micrographs and extracted for 2D classification to generate templates for autopicking of the entire dataset. Reference-free 2D classification was sufficient to remove disordered particles for 3D refinement, although in some cases 3D classification improved the homogeneity of the dataset. Initial models were low-pass filtered to 20–30 Å for the first 3D refinement: WNV (EMD-5296) for WNV_KUN_, bWNV_KUN_ and bMVEV; and DENV-4 (EMD-2485) for bDENV-2. A soft mask excluding the solvent and internal capsid and genome was used to improve alignment. Before subsequent rounds of 3D refinement, the defocus and motion of each particle were refined as well as the astigmatism and anisotropic magnification of the micrograph. For bDENV-2, the 3-fold astigmatism (trefoil) and beam tilt of the dataset were also estimated to improve the resolution of the final reconstruction. The final maps were filtered according to their local resolution using a *B*-factor estimated from the post-processed masked maps.

### Model building

Initial models were produced by the alignment of the reference and target sequences using Clustal Omega^67^ and mapping of the sequence alignment onto an existing structure using Sculptor^68^ from the Phenix suite (v. 1.16)^69^. The 3.1 Å cryo-EM structure of ZIKV (PDB: 6CO8) was used as an initial model. Modelling was performed in Coot (v. 0.8.9.1)^70^ and the models were refined in real space with the phenix.real_space_refine program (PHENIX v. 1.16)^69^ using the secondary structure and non-crystallographic symmetry restraints. The geometry and quality of the models were evaluated using a combination of MolProbity^71^ and PHENIX.

### Automated model building and refinement

To provide an unbiased evaluation of the consistency of the WNV_KUN_ and bWNV_KUN_ reconstructions complementary to the direct map comparison, we performed automatic model building and refinement. A cryo-EM structure of ZIKV (PDB: 6CO8) was used as an initial model (53% sequence identity with WNV_KUN_). Using phenix.map_box, a 4 Å cutout of density corresponding to one M–E dimer was extracted and submitted to Buccaneer (v. 1.6.10)^72^ in the CCP-EM suite (v. 1.4.1)^73^ for five cycles of automated building and refinement. A total of 558 and 566 residues out of 576 were built and refined to an *R*-factor of 0.38 and 0.34 for WNV_KUN_ and bWNV_KUN_, respectively. Automatic building and refinement improved the cross-correlation of the model with the map from 58.9% and 61.6% for the initial model to 78.2% and 81.8% for WNV_KUN_ and bWNV_KUN_, respectively. The resulting models were aligned and the RMSD calculated for each equivalent residue in UCSF Chimera (v. 1.14) and plotted in GraphPad Prism (v. 8.1.2) together with a similar analysis performed on the best refined structures.

### Model analysis

PyMOL (The PyMOL Molecular Graphics System, version 2.3.0; Schrödinger, LLC), UCSF Chimera (v. 1.14)^74^ and UCSF ChimeraX (v. 1.0)^75^ were used to render images of the structures. The Consurf server was used with default parameters and a curated set of genome sequences (Supplementary Table 4) to estimate evolutionary conservation scores and map them onto structures^76^. To compare the maps of bWNV_KUN_ and WNV_KUN_, we utilized the calcfsc function of the program e2proc3d within the EMAN2 software suite (v. 2.3.1)^77^. The results were plotted in GraphPad Prism (v. 8.1.2, La Jolla, CA, USA).

### Reporting summary

Further information on research design is available in the Nature Research Reporting Summary linked to this article.

## Supplementary information

Supplementary Information

Description of Additional Supplementary Files

Supplementary Movie 1

Reporting Summary

## Data Availability

All data associated with this study are presented in the paper, Supplementary files or are available from the corresponding authors on reasonable request. Cryo-EM maps and models have been deposited in the Electron Microscopy Data Bank and the Protein Data Bank, respectively: WNV_KUN_ (PDB ID: 7KVA, EMD-2304); bWNV_KUN_ (PDB ID: 7KV9, EMD-23043); bMVEV (PDB ID: 7KVB, EMD-23045); and bDENV-2 (PDB ID: 7KV8, EMD-23042). Where published structures are mentioned in the text, the PDB accession code is provided. Source data are provided with this paper.

## Source data

Source Data

## References

[1] Sips GJ, Wilschut J, Smit JM (2012). Neuroinvasive flavivirus infections. Rev. Med. Virol..

[2] Blitvich BJ, Firth AE (2015). Insect-specific flaviviruses: a systematic review of their discovery, host range, mode of transmission, superinfection exclusion potential and genomic organization. Viruses.

[3] Pierson TC, Diamond MS (2020). The continued threat of emerging flaviviruses. Nat. Microbiol..

[4] Bhatt S (2013). The global distribution and burden of dengue. Nature.

[5] Deng, S. Q. et al. A review on dengue vaccine development. *Vaccines* **8**, 10.3390/vaccines8010063 (2020). 10.3390/vaccines8010063 PMC7159032 32024238

[6] Sejvar, J. J. West Nile virus infection. *Microbiol. Spectr.* **4**, 10.1128/microbiolspec.EI10-0021-2016 (2016). 10.1128/microbiolspec.EI10-0021-2016 27337465

[7] Young, P. R. in *Dengue and Zika: Control and Antiviral Treatment Strategies*, Vol. Advances in Experimental Medicine and Biology (eds Hilgenfeld, R. & Vasudevan, S.) (Singapore, 2018).

[8] Song BH, Yun SI, Woolley M, Lee YM (2017). Zika virus: history, epidemiology, transmission, and clinical presentation. J. Neuroimmunol..

[9] Therkelsen MD (2018). Flaviviruses have imperfect icosahedral symmetry. Proc. Natl Acad. Sci. USA.

[10] Kuhn RJ (2002). Structure of dengue virus. Cell.

[11] Pierson TC, Diamond MS (2012). Degrees of maturity: the complex structure and biology of flaviviruses. Curr. Opin. Virol..

[12] Yu IM (2008). Structure of the immature dengue virus at low pH primes proteolytic maturation. Science.

[13] Li L (2008). The flavivirus precursor membrane-envelope protein complex: structure and maturation. Science.

[14] Heinz FX, Stiasny K (2012). Flaviviruses and flavivirus vaccines. Vaccine.

[15] Barba-Spaeth G (2016). Structural basis of potent Zika-dengue virus antibody cross-neutralization. Nature.

[16] Lok SM (2008). Binding of a neutralizing antibody to dengue virus alters the arrangement of surface glycoproteins. Nat. Struct. Mol. Biol..

[17] Dejnirattisai W (2015). A new class of highly potent, broadly neutralizing antibodies isolated from viremic patients infected with dengue virus. Nat. Immunol..

[18] Teoh EP (2012). The structural basis for serotype-specific neutralization of dengue virus by a human antibody. Sci. Transl. Med..

[19] Fibriansah G (2015). A highly potent human antibody neutralizes dengue virus serotype 3 by binding across three surface proteins. Nat. Commun..

[20] Fibriansah G (2014). A potent anti-dengue human antibody preferentially recognizes the conformation of E protein monomers assembled on the virus surface. EMBO Mol. Med..

[21] Qiu X (2018). Structural basis for neutralization of Japanese encephalitis virus by two potent therapeutic antibodies. Nat. Microbiol..

[22] Kaufmann B (2010). Neutralization of West Nile virus by cross-linking of its surface proteins with Fab fragments of the human monoclonal antibody CR4354. Proc. Natl Acad. Sci. USA.

[23] Sevvana M (2018). Refinement and analysis of the nature Zika virus Cryo-EM structure at 3.1 A resolution. Structure.

[24] Hobson-Peters J (2019). A recombinant platform for flavivirus vaccines and diagnostics using chimeras of a new insect-specific virus. Sci. Transl. Med..

[25] Harrison, J. J. et al. Antigenic characterization of new lineage II insect-specific flaviviruses in Australian mosquitoes and identification of host restriction factors. *mSphere* **5**, 10.1128/mSphere.00095-20 (2020). 10.1128/mSphere.00095-20 PMC7300350 32554715

[26] Vet, L. J. et al. Protective efficacy of a chimeric insect-specific flavivirus vaccine against West Nile virus. *Vaccines* **8**, 10.3390/vaccines8020258 (2020). 10.3390/vaccines8020258 PMC7349994 32485930

[27] Frost MJ (2012). Characterization of virulent West Nile virus Kunjin strain, Australia, 2011. Emerg. Infect. Dis..

[28] Doherty RL, Carley JG, Mackerras MJ, Marks EN (1963). Studies of arthropod-borne virus infections in Queensland. III. Isolation and characterization of virus strains from wild-caught mosquitoes in North Queensland. Aust. J. Exp. Biol. Med. Sci..

[29] Rey FA, Heinz FX, Mandl C, Kunz C, Harrison SC (1995). The envelope glycoprotein from tick-borne encephalitis virus at 2 A resolution. Nature.

[30] Kanai R (2006). Crystal structure of west nile virus envelope glycoprotein reveals viral surface epitopes. J. Virol..

[31] Nybakken GE, Nelson CA, Chen BR, Diamond MS, Fremont DH (2006). Crystal structure of the West Nile virus envelope glycoprotein. J. Virol..

[32] Cherrier MV (2009). Structural basis for the preferential recognition of immature flaviviruses by a fusion-loop antibody. EMBO J..

[33] Walker PJ (2019). Changes to virus taxonomy and the International Code of Virus Classification and Nomenclature ratified by the International Committee on Taxonomy of Viruses (2019). Arch. Virol..

[34] Knox J (2012). Murray Valley encephalitis: a review of clinical features, diagnosis and treatment. Med. J. Aust..

[35] Wang X (2017). Near-atomic structure of Japanese encephalitis virus reveals critical determinants of virulence and stability. Nat. Commun..

[36] Hobson-Peters J (2008). A glycosylated peptide in the West Nile virus envelope protein is immunogenic during equine infection. J. Gen. Virol..

[37] Beasley DW (2005). Envelope protein glycosylation status influences mouse neuroinvasion phenotype of genetic lineage 1 West Nile virus strains. J. Virol..

[38] Shirato K (2004). Viral envelope protein glycosylation is a molecular determinant of the neuroinvasiveness of the New York strain of West Nile virus. J. Gen. Virol..

[39] Goto A (2005). Role of the N-linked glycans of the prM and E envelope proteins in tick-borne encephalitis virus particle secretion. Vaccine.

[40] Zhang Y, Chen P, Cao R, Gu J (2011). Mutation of putative N-linked glycosylation sites in Japanese encephalitis virus premembrane and envelope proteins enhances humoral immunity in BALB/C mice after DNA vaccination. Virol. J..

[41] Hanna SL (2005). N-linked glycosylation of west nile virus envelope proteins influences particle assembly and infectivity. J. Virol..

[42] Davis CW (2006). West Nile virus discriminates between DC-SIGN and DC-SIGNR for cellular attachment and infection. J. Virol..

[43] Yun SI (2014). A molecularly cloned, live-attenuated japanese encephalitis vaccine SA14-14-2 virus: a conserved single amino acid in the ij Hairpin of the Viral E glycoprotein determines neurovirulence in mice. PLoS Pathog..

[44] Watterson D, Kobe B, Young PR (2012). Residues in domain III of the dengue virus envelope glycoprotein involved in cell-surface glycosaminoglycan binding. J. Gen. Virol..

[45] Lee JW, Chu JJ, Ng ML (2006). Quantifying the specific binding between West Nile virus envelope domain III protein and the cellular receptor alphaVbeta3 integrin. J. Biol. Chem..

[46] Beigel JH (2010). Safety and pharmacokinetics of single intravenous dose of MGAWN1, a novel monoclonal antibody to West Nile virus. Antimicrob. Agents Chemother..

[47] Zhang X (2013). Cryo-EM structure of the mature dengue virus at 3.5-A resolution. Nat. Struct. Mol. Biol..

[48] DiNunno NM (2020). Identification of a pocket factor that is critical to Zika virus assembly. Nat. Commun..

[49] Renner, M., Dejnirattisai, W., Carrique, L. et al. Flavivirus maturation leads to the formation of an occupied lipid pocket in the surface glycoproteins. *Nat Commun.* **12**, 1238 (2021). 10.1038/s41467-021-21505-9 PMC7902656 33623019

[50] Kostyuchenko VA, Zhang Q, Tan JL, Ng TS, Lok SM (2013). Immature and mature dengue serotype 1 virus structures provide insight into the maturation process. J. Virol..

[51] Sirohi D (2016). The 3.8 A resolution cryo-EM structure of Zika virus. Science.

[52] Kostyuchenko VA (2016). Structure of the thermally stable Zika virus. Nature.

[53] Heinz FX (1994). Structural changes and functional control of the tick-borne encephalitis virus glycoprotein E by the heterodimeric association with protein prM. Virology.

[54] Smith T (1986). The site of attachment in human rhinovirus 14 for antiviral agents that inhibit uncoating. Science.

[55] Casanova V, Sousa FH, Stevens C, Barlow PG (2018). Antiviral therapeutic approaches for human rhinovirus infections. Fut. Virol..

[56] Yant SR (2019). A highly potent long-acting small-molecule HIV-1 capsid inhibitor with efficacy in a humanized mouse model. Nat. Med..

[57] Chen L (2018). Implication for alphavirus host-cell entry and assembly indicated by a 3.5A resolution cryo-EM structure. Nat. Commun..

[58] Haag L (2002). Acid-induced movements in the glycoprotein shell of an alphavirus turn the spikes into membrane fusion mode. EMBO J..

[59] Rouvinski A (2017). Covalently linked dengue virus envelope glycoprotein dimers reduce exposure of the immunodominant fusion loop epitope. Nat. Commun..

[60] Slon-Campos JL (2019). A protective Zika virus E-dimer-based subunit vaccine engineered to abrogate antibody-dependent enhancement of dengue infection. Nat. Immunol..

[61] Reed, L. J. & Muench, H. A simple method of estimating fifty percent endpoints. *Am. J. Hyg.* **27**, 493–497 (1938).

[62] Clark DC (2007). In situ reactions of monoclonal antibodies with a viable mutant of Murray Valley encephalitis virus reveal an absence of dimeric NS1 protein. J. Gen. Virol..

[63] Piyasena TBH (2017). Infectious DNAs derived from insect-specific flavivirus genomes enable identification of pre- and post-entry host restrictions in vertebrate cells. Sci. Rep..

[64] Zheng SQ (2017). MotionCor2: anisotropic correction of beam-induced motion for improved cryo-electron microscopy. Nat. Methods.

[65] Zhang K (2016). Gctf: real-time CTF determination and correction. J. Struct. Biol..

[66] Zivanov J, Nakane T, Scheres SHW (2020). Estimation of high-order aberrations and anisotropic magnification from cryo-EM data sets in RELION-3.1. IUCrJ.

[67] Sievers F, Higgins DG (2018). Clustal Omega for making accurate alignments of many protein sequences. Protein Sci..

[68] Bunkoczi G, Read RJ (2011). Improvement of molecular-replacement models with Sculptor. Acta Crystallogr. D.

[69] Adams PD (2010). PHENIX: a comprehensive Python-based system for macromolecular structure solution. Acta Crystallogr. Sect. D.

[70] Emsley P, Lohkamp B, Scott WG, Cowtan K (2010). Features and development of Coot. Acta Crystallogr. Sect. D.

[71] Williams CJ (2018). MolProbity: more and better reference data for improved all-atom structure validation. Protein Sci..

[72] Cowtan K (2006). The Buccaneer software for automated model building. 1. Tracing protein chains. Acta Crystallogr. D.

[73] Winn MD (2011). Overview of the CCP4 suite and current developments. Acta Crystallogr. D.

[74] Pettersen EF (2004). UCSF Chimera—a visualization system for exploratory research and analysis. J. Comput. Chem..

[75] Goddard TD (2018). UCSF ChimeraX: meeting modern challenges in visualization and analysis. Protein Sci..

[76] Ashkenazy H (2016). ConSurf 2016: an improved methodology to estimate and visualize evolutionary conservation in macromolecules. Nucleic Acids Res..

[77] Tang G (2007). EMAN2: an extensible image processing suite for electron microscopy. J. Struct. Biol..

