# Supplementary material for: A unified route for flavivirus structures uncovers essential pocket factors conserved across pathogenic viruses
Source: Nat Commun. 2021 Jun 1;12:3266. doi: 10.1038/s41467-021-22773-1 (PMC8169900; doi:10.1038/s41467-021-22773-1)
Supplement: Supplementary file 1 — Supplementary Information [file 41467_2021_22773_MOESM1_ESM.pdf]

A unified route for flavivirus structures uncovers essential pocket factors conserved across pathogenic viruses

Joshua M Hardy[1,†], Natalee D Newton[2,†], Naphak Modhiran[2], Connor AP Scott[2], Hari Venugopal[3], Laura J Vet[2], Paul R Young[2], Roy A Hall[2], Jody Hobson-Peters[2], Fasséli Coulibaly[1,†*], Daniel Watterson[2,†*]

1. Infection & Immunity Program, Biomedicine Discovery Institute & Department of Biochemistry and Molecular Biology, Monash University, Clayton, VIC, Australia
2. Australian Infectious Diseases Research Centre, School of Chemistry and Molecular Biosciences, The University of Queensland, Brisbane, Australia
3. Ramaciotti Centre for Cryo-Electron Microscopy, Monash University, Clayton, VIC, Australia

* Correspondence to: fasseli.coulibaly@monash.edu , d.watterson@uq.edu.au

† These authors contributed equally

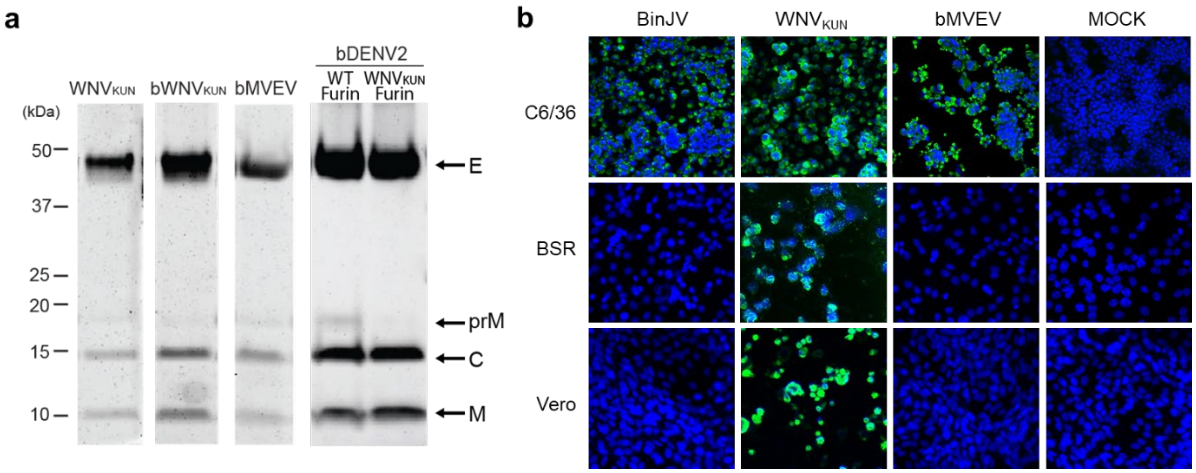

Supplementary Figure 1. Characterisation of BinJV chimeras. (a) SDS-PAGE (4-12%) analysis of gradient purified WNV_{KUN}, $bWNV_{KUN}$, bMVEV and bDENV2 (WT and enhanced furin cleavage site) virions stained with SYPRO Ruby protein stain. Viral protein identity indicated by arrows. **(b)** Analysis of bMVEV growth in vertebrate cells. C6/36, BSR and Vero cell monolayers were infected at an MOI of 1 or mock-infected and fixed after 7 days incubation. IFA analysis was performed using the pan-flavivirus anti-NS1 antibody, 4G4. Nuclei were stained with Hoechst 33342. Images taken at 20x magnification.

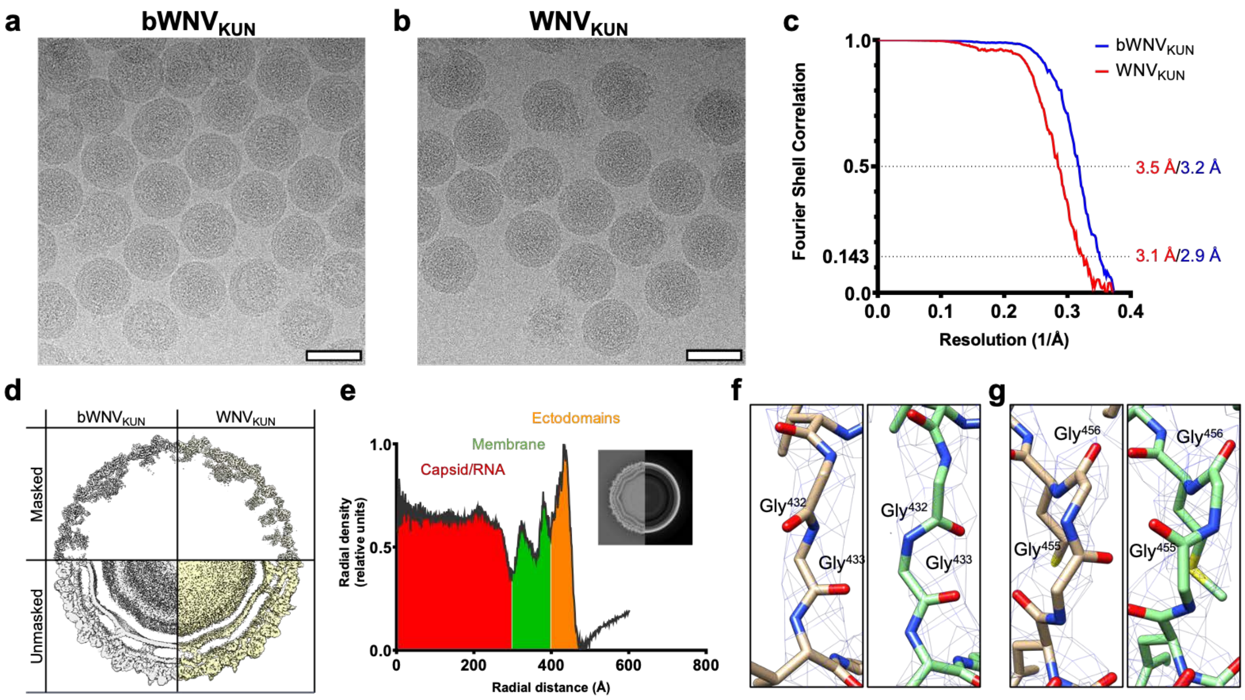

Supplementary Figure 2. Cryo-EM reconstructions of WNV_{KUN} and $bWNV_{KUN}$. (a-b) Typical micrographs for $bWNV_{KUN}$ (a) and WNV_{KUN} (b). Scale bar represents 50 nm. **(c)** Fourier shell correlation plots for $bWNV_{KUN}$ (blue) and WNV_{KUN} (red) reconstructions. **(d)** Comparison of masked and unmasked maps of $bWNV_{KUN}$ and WNV_{KUN}. **(e)** Radial average of $bWNV_{KUN}$ map coloured according to composition: capsid/RNA (red), membrane (green), and protein ectodomains (orange). Inset shows central slice of reconstruction (left) and the radially averaged image (right). Refinement of atomic models were performed independently for $bWNV_{KUN}$ (beige sticks) and WNV_{KUN} (green sticks) in the EM maps (blue mesh). The most significant differences were mainchain flips of two glycine residues, **(f)** Gly432 and **(g)** Gly455.

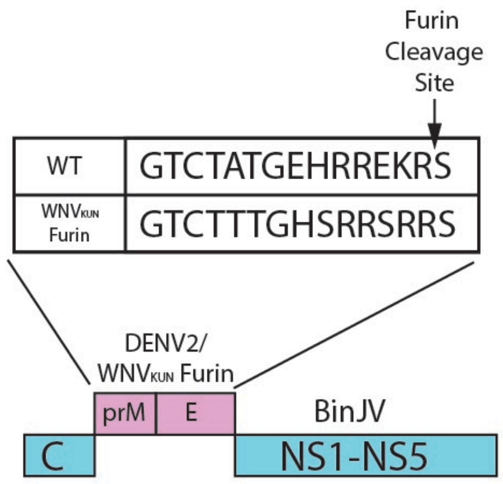

Supplementary Figure 3. Optimization of the DENV Furin cleavage site. Residues surrounding the furin cleavage site in bDENV-2 were exchanged with those of WNV_{KUN} to improve prM processing and particle homogeneity. Sequence alignment is shown with furin site indicated.

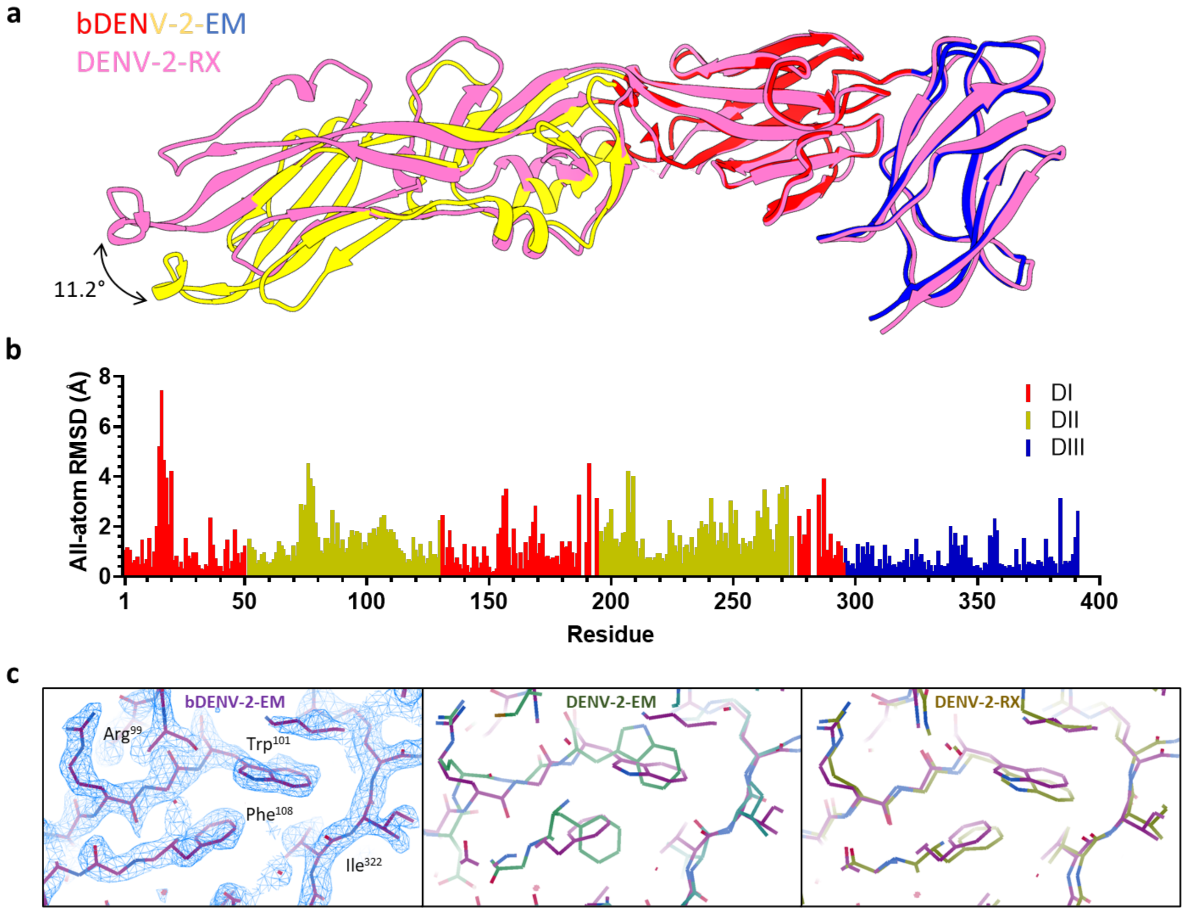

Supplementary Figure 4. Comparison of bDENV-2 structure to a crystal structure of DENV-2. (a) Alignment of the ectodomain of E5 between bDENV-2 (colored by domain) and a crystal structure of DENV-2 (pink, PDB: 4UTC) in a cartoon representation. An arrow indicates the hinge movement of DII. **(b)** All-atom RMSD between the structures in (a) coloured by domains. Domains were aligned independently of each other. **(c)** Comparison of the bDENV-2 structure (purple) with an EM structures of DENV-2 (green, PDB: 3J27) and a crystal structure of DENV-2 (yellow, PDB: 4UTC) near the fusion loop. Each structure is displayed as sticks with bDENV-2 present in each panel.

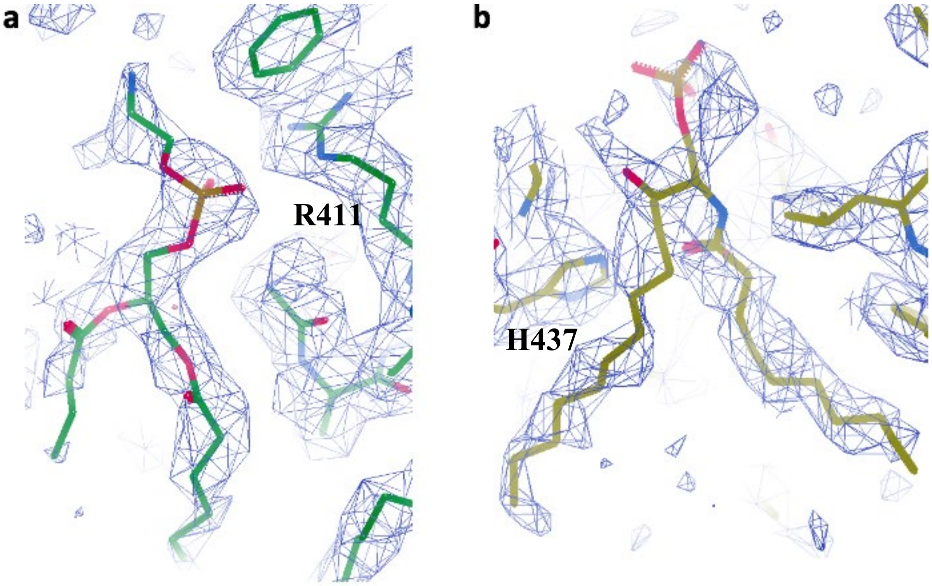

Supplementary Figure 5. Pocket factors in the bDENV-2 structure. Density fit of site 1 (**a**) and site 2 (**b**) pocket factors modelled as a phosphatidylethanolamine (PDB ligand 6OU) and phosphoceramide (PBD ligand 1Q0), respectively. The density is shown at 1.3 sigma. While the quality of the map does not allow unambiguous identification of the pocket factors, very similar bifurcated densities are observed for the 3 independent molecules in the asymmetric unit of the icosahedra particle.

Supplementary Table 1. Cryo-EM data collection, refinement and validation statistics

	WNV_{KUN} (EMD-23044, PDB 7KVA)	$bWNV_{KUN}$ (EMD-23043, PDB 7KV9)	bMVEV (EMD-23045, PDB 7KVB)	bDENV2 (EMD-23042, PDB 7KV8)
**Data collection and processing**				
Magnification	105,000	105,000	130,000	130,000
Voltage (kV)	300	300	300	300
Electron exposure ($e^{-}/Å^2$)	57.6	30.6	25	67.7
Defocus range (μm)	0.5-1.8	0.3-1.7	0.6-2.0	0.3-2.0
Pixel size (Å)	0.67	0.67	0.52	0.52
Micrographs	1,517	3,209	2,829	4,722
Symmetry imposed	I	I	I	I
Initial particle images (no.)	33,918	130,675	13,143	196,292
Final particle images (no.)	7,551	40,058	12,812	111,223
Map resolution at 0.143 FSC (Å)	3.1	2.9	3.7	2.5
**Refinement**				
Initial model used (PDB code)	($bWNV_{KUN}$)	6CO8	($bWNV_{KUN}$)	6CO8
Model resolution (Å)	2.9/3.1	2.7/2.9	3.3/3.6	2.2/2.4
FSC threshold	0.143/0.5	0.143/0.5	0.143/0.5	0.143/0.5
Cross-correlation (masked)	0.82	0.85	0.80	0.84
Map-sharpening *B* factor ($Å^2$)	-82	-98	-128	-77
Model composition				
Nonhydrogen	12877	12893	12717	13780
Protein residues	1716	1716	1716	1701
Ligands	NAG: 3	NAG: 3	NAG: 3	NAG:3<br>1Q0:3<br>6OU:3
Water	-	-	-	454
B factors ($Å^2$)				
Protein	9.25	17.56	15.53	10.16
Ligand	21.85	29.25	33.65	22.36
Water	-	-	-	7.07
R.m.s. deviations				
Bond lengths (Å)	0.002	0.003	0.002	0.008
Bond angles (°)	0.467	0.481	0.445	0.779
Validation				
MolProbity score	1.35	1.33	1.54	1.62
Clashscore	6.38	5.54	7.63	8.70
Poor rotamers (%)	0.00	0.00	0.07	1.26
Ramachandran plot				
Favored (%)	98.53	97.89	97.36	97.62
Allowed (%)	1.47	2.11	2.64	2.38
Disallowed (%)	0.00	0.00	0.00	0.00

Supplementary Table 2. Primers used for chimeric virus generation

Fragment	Template	Primer (5' – 3')	
5' UTR – C	BinJV	F	GCCCGCAACGATCTGGTAAACAGTATATTTTGCGTGTGCG
		R	AGCCATCGCTCCTGCTCCAACGAGCAGC
prM – E	WNV_{KUN}	F	GCTGCTCGTTGGAGCAGGAGCGATGGCTGTCACTCTCTCCAACTT CCAAGGGAAGG
		R	CCAGACTGCATCCTATTTCCGATAGAGCATGCACGTTCACGGAAA GAAAGAGC
	MVEV	F	GCTGCTCGTTGGAGCAGGAGCGATGGCTTTAAAGCTTTCCACCTT CCAGGGC
		R	CCAGACTGCATCCTATTTCCGATAGAGCATGGACATTTGTGGCCA GG
	DENV-2	F	GCTGCTCGTTGGAGCAGGAGCGATGGCTTTTCATCTGACCACACG TAACGGAGAA
		R	CTTTTCGGCTAATGTCCAGACTGCATCCTATTTCGGCCTGCACCAT AACTCCCAAATAC
	DENV-2 Furin site	F	ACAGGACACTCAAGACGCAGTAGAAGGTCAGTGGCACTCGTTCC ACATGTGGGAA
		R	TGCCACTGACCTTCTACTGCGTCTTGAGTGTCCTGTGGTGGTACA CGTCCCATAAGT
NS1 – 2B	BinJV	F	CTATCGGAAATAGGATGCAGTCTGGACATTAGCCGAAAAG
		R	CCACAACACAGTCCCCCGCTTGTTTGATTT
NS3 – 4B	BinJV	F	AAATCAAACAAGCGGGGGACTGTGTTGTGG
		R	GGTGGCCTGTAATCCCCTCCTAGGAACTCC
NS5	BinJV	F	GGAGTTCCTAGGAGGGGATTACAGGCCACC
		R	TGCCATGCCGACCCAGATACTTGATGTTTC
3' UTR	BinJV	F	GGCAATGTGATCTAAGGATCTACGAACGAG
		R	TGCCATGCCGACCCAGATACTTGATGTTTC
Linker	BinJV	F	TGGATTGGGGATTGAGAAACATCAAGTATCTGGGTCGGCATGGC ATCTCCACCTCCTCGC
		R	GTGTTTTGAAACGCACACGCAAAATATACTGTTTACCAGATCGTT GCGGGCTGTATTTATAGGC
**bDENV2 lipid mutagenesis primers**			
R441A		F	GACAACAATGAGGGGAGCGAAGGCAATGGCCATTTTAGGTGACA CAGCTTGGGATTTTG
		R	CACCTAAAATGGCCATTGCCTTCGCTCCCCTCATTGTTGTCTCAAT CATTTGGCCGATAG
R411K		F	GACAACAATGAGGGGAGCGAAGAAAATGGCCATTTTAGGTGACA CAGCTTGGGATTTTG
		R	CACCTAAAATGGCCATTTTCTTCGCTCCCCTCATTGTTGTCTCAAT CATTTGGCCGATAG
R411D		F	GACAACAATGAGGGGAGCGAAGGATATGGCCATTTTAGGTGACA CAGCTTGGGATTTTG
		R	CACCTAAAATGGCCATATCCTTCGCTCCCCTCATTGTTGTCTCAAT CATTTGGCCGATAG
W420A		F	CCATTTTAGGTGACACAGCTGCAGATTTTGGATCCCTGGGAGGAG TGTTTACATCTATAG

	R	CTCCTCCCAGGGATCCAAAATCTGCAGCTGTGTCACCTAAAATGG CCATTCTCTTCGCTC
F422A	F	CATTTTAGGTGACACAGCTTGGGATGCAGGATCCCTGGGAGGAG TGTTTACATCTATAGG
	R	CTCCTCCCAGGGATCCTGCATCCCAAGCTGTGTCACCTAAAATGG CCATTCTCTTCGCTC
H437A	F	GTTTACATCTATAGGAAAGGCTCTCGCACAAGTTTTCGGAGCAAT CTATGGGGCTGCC
	R	GCTCCGAAAACTTGTGCGAGAGCCTTTCCTATAGATGTAAACACT CCTCCCAGGGATC
G441A	F	GAAAGGCTCTCCACCAAGTTTTCGCAGCAATCTATGGGGCTGCCT TCAGTGGGGTCTC
	R	CAGCCCCATAGATTGCTGCGAAAACTTGGTGGAGAGCCTTTCCTA TAGATGTAAACACTC
Y444A	F	CTCCACCAAGTTTTCGGAGCAATCGCAGGGGCTGCCTTCAGTGGG GTCTCATGGACTATG
	R	CACTGAAGGCAGCCCCTGCGATTGCTCCGAAAACTTGGTGGAGA GCCTTTCCTATAGATG
F448A	F	GTTTTCGGAGCAATCTATGGGGCTGCCGCAAGTGGGGTCTCATGG ACTATGAAAATCCTC
	R	GAGACCCCACTTGCGGCAGCCCCATAGATTGCTCCGAAAACTTGG TGGAGAGCCTTTC
L489A	F	GTCACTAGTATTGGTGGGAGTCGTGACGCTGTATGCAGGAGTTAT GGTGCAGGCCGAAATAGGATGCAGTCTGGACATTAGCCGAAAAG
	R	CTTTTCGGCTAATGTCCAGACTGCATCCTATTTCGGCCTGCACCAT AACTCCTGCATACAGCGTCACGACTCCCACCAATACTAGTGAC

Supplementary Table 3. Vitrification parameters for plunge freezing.

Sample	Wait time (s)	Blot time (s)	Blot force	Drain time (s)
bDENV-2	0	2.5	-2	1
bMVEV	0	2	-10	1
$bWNV_{KUN}$	0	2	-12	1
WNV_{KUN}	0	2	-10	1

Supplementary Table 4. Sequences used for conservation analysis. For mosquito-borne flaviviruses (MBF's) and tick-borne flaviviruses (TBF's) genomes were obtained from the Swiss-Prot database. For species with several strains, only one strain was selected. In the case of the dual-host-affiliated insect-specific flaviviruses (dISF's) the Binjari genome was used to search the UniRef90 database for related genomes and entries which had complete genomes were included.

Entry	Entry name	Organism	Length	Class
Q91B85	POLG_ALKV	Alkhumra hemorrhagic fever virus (ALKV)	3416	TBF
D7RF80	POLG_KFDV	Kyasanur forest disease virus (KFDV)	3416	TBF
P29837	POLG_LANVT	Langat virus (strain TP21)	3414	TBF
P22338	POLG_LIV	Louping ill virus (Li)	3414	TBF
Q7T6D2	POLG_OHFV	Omsk hemorrhagic fever virus (OHFV)	3414	TBF
Q04538	POLG_POWVL	Tick-borne powassan virus (strain LB) (POWV) (Powassan virus)	3415	TBF
C8XPA8	POLG_BANV	Banzi virus (BANV)	3393	MBF
Q32ZE0	POLG_BUSV	Bussuquara virus (BUSV)	3429	MBF
P17763	POLG_DEN1W	Dengue virus type 1 (strain Nauru/West Pac/1974) (DENV-1)	3392	MBF
P14340	POLG_DEN2N	Dengue virus type 2 (strain Thailand/NGS-C/1944) (DENV-2)	3391	MBF
P27915	POLG_DEN3P	Dengue virus type 3 (strain Philippines/H87/1956) (DENV-3)	3390	MBF
Q58HT7	POLG_DEN4P	Dengue virus type 4 (strain Philippines/H241/1956) (DENV-4)	3387	MBF
C8XPB2	POLG_EHV	Edge Hill virus (EHV)	3401	MBF
Q32ZD7	POLG_ILHV	Ilheus virus (ILHV)	3424	MBF
P27395	POLG_JAEV1	Japanese encephalitis virus (strain SA-14) (JEV)	3432	MBF
Q32ZD5	POLG_KOKV	Kokobera virus (KOKV)	3410	MBF
P14335	POLG_KUNJM	Kunjin virus (strain MRM61C)	3433	MBF
P05769	POLG_MVEV5	Murray valley encephalitis virus (strain MVE-1-51) (MVEV)	3434	MBF
Q32ZD4	POLG_ROCV	Rocio virus (ROCV)	3425	MBF
P09732	POLG_STEVM	St. louis encephalitis virus (strain MS1-7)	3412	MBF
Q5WPU5	POLG_USUV	Usutu virus (USUV)	3434	MBF
C5H431	POLG_WSLV	Wesselsbron virus (WSLV)	3405	MBF
Q9Q6P4	POLG_WNV9	West Nile virus (strain NY-99) (WNV)	3433	MBF
Q6DV88	POLG_YEFVA	Yellow fever virus (strain Ghana/Asibi/1927) (YFV)	3411	MBF
Q32ZE1	POLG_ZIKV	Zika virus (ZIKV)	3419	MBF
A0A5K6VMX2	A0A5K6VMX2_9FLAV	Binjari virus	3433	dISF
H9CH24	H9CH24_9FLAV	Chaoyang virus	3435	dISF
H9BYJ9	H9BYJ9_9FLAV	Donggang virus	3444	dISF
A0A5B8RNL2	A0A5B8RNL2_9FLAV	Guapiacu virus	3438	dISF
A0A088BPV6	A0A088BPV6_9FLAV	Lammi virus	3434	dISF
A0A292E049	A0A292E049_9FLAV	Long Pine Key virus	3459	dISF
A0A290Y5I2	A0A290Y5I2_9FLAV	Marisma mosquito virus	3450	dISF

Supplementary Note 1. UTR and OpiE2 linker region for BinJV chimeras. The 3’ (red) and 5’ (blue) UTRs of BinJV are connected during CPER via a linker (black) that includes an *Orgyia pseudotsugata* multicapsid nucleopolyhedrosis virus immediate-early 2 (OpIE2) promoter and hepatitis delta virus ribozyme-poly A (HDVr-pA) site.

GGATCTACGAACGAGAATAAGTAGAAGAACGGAACGACAGAGTCAGGCCTCAAATGAGCCAGCA
TTAATGAGAGTAAGTGCTGCTGCCTGTGCCTCTCCTTAACACGTGGTAGCGCCACTCGTGTTTCGTT
ACCTAATAGCGCTAGAGTCAGACCCAAGTAGGCCAGGGCTATGGTTGTAAGCCCTGCTGTCTGTG
GCAGCCATCCAGTGGTAATGCGTCGCACCACTAAGGATTAATAGACGTATATTGGGAGGGACTGG
TGAGGAGCAGCAAGCTCGAGCTGCATCACCCACTGGTACTATCGGTTAGAGGAAACCCCCTCCAA
AATGTAGAGCATCATATCGACACCTGGGAAAGACCGGAGATACCTCTTGCTTCACAGCACTCAAT
CCACAAGGCACAGATCGCCGAATAATTGTGGATTGGGGATTGAGAAACATCAAGTATCTGGGTCG
GCATGGCATCTCCACCTCCTCGCGGTCCGACCTGGGCATCCGAAGGAGGACGTCGTCCACTCGGAT
GGCTAAGGGAGAGCCACTTTTCTCTCGATTCTCTATCGGAATCTAGGGAGCTCGGATCCAGACATG
ATAAGATACATTGATGAGTTTGGACAAACCACAACTAGAATGCAGTGAAAAAAATGCTTTATTTG
TGAAATTTGTGATGCTATTGCTTTATTTGTAACCATTATAAGCTGCAATAAACAAGTTAACAACAA
CAATTGCTCGAGGGGGGGCCCGGTACCTTGAAGCTGTCCCTGATGGTCGTCATCTACCTGCCTGGA
CAGCATGGCCTGCAACGCGGGCATCCCGATGCCGCCGGAAGCGAGAAGAATCATAATGGGGAAG
GCCATCCAGCCTCGCGTCGGCGCTTAAGGATCATGATGATAAACAATGTATGGTGCTAATGTTGCT
TCAACAACAATTCTGTTGAACTGTGTTTTCATGTTTGCCAACAAGCACCTTTATACTCGGTGGCCTC
CCCACCACCAACTTTTTTGCACTGCAAAAAAACACGCTTTTGCACGCGGGCCCATACATAGTACAA
ACTCTACGTTTCGTAGACTATTTTACATAAATAGTCTACACCGTTGTATACGCTCCAAATACACTAC
CACACATTGAACCTTTTTGCAGTGCAAAAAAGTACGTGTCGGCAGTCACGTAGGCCGGCCTTATCG
GGTCGCGTCCTGTCACGTACGAATCACATTATCGGACCGGACGAGTGTTGTCTTATCGTGACAGGA
CGCCAGCTTCCTGTGTTGCTAACCGCAGCCGGACGCAACTCCTTATCGGAACAGGACGCGCCTCCA
TATCAGCCGCGCGTTATCTCATGCGCGTGACCGGACACGAGGCGCCCGTCCCGCTTATCGCGCCTA
TAAATACAGCCCGCAACGATCTGGTAAACAGTATATTTTGCGTGTGCGTTTCAAAACACAGATTGT
ATTGAAAGTACGTAAGAGTATAACACGTTGGAATAAAGTTTTGGATCAAGAGGAAAATC
