# Supplementary material for: A unified route for flavivirus structures uncovers essential pocket factors conserved across pathogenic viruses
Source: Nat Commun. 2021 Jun 1;12:3266. doi: 10.1038/s41467-021-22773-1 (PMC8169900; doi:10.1038/s41467-021-22773-1)
Supplement: Supplementary file 2 — Description of Additional Supplementary Files [file 41467_2021_22773_MOESM2_ESM.pdf]

Description of Additional Supplementary Files

File Name: Supplementary Movie 1
Description: **The structure of bDENV-2.** Representation of the mature bDENV2 flavivirus as in Fig. 3c. The asymmetric unit consists of 3 copies of the M-E complex arranged in a characteristic herringbone pattern. Density for the water molecules is represented in red with the lipid density displayed in yellow and green. A single M-E subunit is colored by domain as in Fig. 8a and zooms show the lipid molecules enclosed with the stem of E.
