# Supplementary material for: A unified route for flavivirus structures uncovers essential pocket factors conserved across pathogenic viruses
Source: Nat Commun. 2021 Jun 1;12:3266. doi: 10.1038/s41467-021-22773-1 (PMC8169900; doi:10.1038/s41467-021-22773-1)
Supplement: Supplementary file 4 — Reporting Summary [file 41467_2021_22773_MOESM4_ESM.pdf]

nature research

Corresponding author(s): Daniel Watterson

Last updated by author(s): Mar 16, 2021

Reporting Summary

Nature Research wishes to improve the reproducibility of the work that we publish. This form provides structure for consistency and transparency in reporting. For further information on Nature Research policies, see our Editorial Policies and the Editorial Policy Checklist.

Statistics

For all statistical analyses, confirm that the following items are present in the figure legend, table legend, main text, or Methods section.

n/a	Confirmed	
☒	☐	The exact sample size (*n*) for each experimental group/condition, given as a discrete number and unit of measurement
☒	☐	A statement on whether measurements were taken from distinct samples or whether the same sample was measured repeatedly
☒	☐	The statistical test(s) used AND whether they are one- or two-sided<br>*Only common tests should be described solely by name; describe more complex techniques in the Methods section.*
☒	☐	A description of all covariates tested
☒	☐	A description of any assumptions or corrections, such as tests of normality and adjustment for multiple comparisons
☒	☐	A full description of the statistical parameters including central tendency (e.g. means) or other basic estimates (e.g. regression coefficient) AND variation (e.g. standard deviation) or associated estimates of uncertainty (e.g. confidence intervals)
☒	☐	For null hypothesis testing, the test statistic (e.g. *F*, *t*, *r*) with confidence intervals, effect sizes, degrees of freedom and *P* value noted<br>*Give P values as exact values whenever suitable.*
☒	☐	For Bayesian analysis, information on the choice of priors and Markov chain Monte Carlo settings
☒	☐	For hierarchical and complex designs, identification of the appropriate level for tests and full reporting of outcomes
☒	☐	Estimates of effect sizes (e.g. Cohen's *d*, Pearson's *r*), indicating how they were calculated

Our web collection on statistics for biologists contains articles on many of the points above.

Software and code

Policy information about availability of computer code

Data collection	Cryo-EM movies recorded using EPU 2 (FEI)
Data analysis	RELION 3.1, MotionCor2 v. 1.1.0, Gctf v. 1.06, Phenix suite v 1.16, Coot (v. 0.8.9.1), MolProbity 4.5.1, Buccaneer (v. 1.6.10)<br>CCP-EM (v. 1.4.1), UCSF Chimera (v. 1.14), GraphPad Prism (v. 8.1.2), PyMOL (v. 2.3.0), UCSF ChimeraX (v. 1.0), EMAN2 (v. 2.3.1)

For manuscripts utilizing custom algorithms or software that are central to the research but not yet described in published literature, software must be made available to editors and reviewers. We strongly encourage code deposition in a community repository (e.g. GitHub). See the Nature Research guidelines for submitting code & software for further information.

Data

Policy information about availability of data

All manuscripts must include a data availability statement. This statement should provide the following information, where applicable:

- Accession codes, unique identifiers, or web links for publicly available datasets
- A list of figures that have associated raw data
- A description of any restrictions on data availability

The data supporting the findings of this manuscript are available from the corresponding authors upon reasonable request. A Reporting Summary for this Article is available as a Supplementary Information file. Source data are provided with this paper. Cryo-EM maps and models have been deposited in the Electron Microscopy Data Bank and the Protein Data Bank, respectively: WNVKUN (PDB ID: 7KVA, EMD-23044); bWNVKUN (PDB ID: 7kV9, EMD-23043); bMVEV (PDB ID: 7KVB, EMD-23045); and bDENV-2 (PDB ID: 7KV8, EMD-23042). Where published structures are mentioned in the text, the PDB code is provided.
Fig2 includes all four structures WNVKUN, bWNVKUN, bDENV2, bMVEV. Validation reports attached.
Fig3 included analysis of WNVKUN and bWNVKUN structures
Fig4 includes bMVEV, bWNVKUN and published JEV structure PDB:5WSN

Fig5 includes bMVEV, bWNVKUN and published JEV structure PDB:5WSN
Fig6 includes bMVEV, bWNVKUN and published WNV structure PDB: 1ZTX
Fig8 includes bDENV-2 and published structures: DENV-2, PDB: 3J27 and PDB: 3C5X; ZIKV, PDB: 6CO8 and EMD-7543; TBEV, PDB: 5O6A and EMD-3752.
Fig9 includes bDENV-2 and published DENV-2 structure: PDB: 4B03
Supplementary Figure 4 includes bDENV-2 and published DENV-2 structures PDB: 3J27 and PDB: 4UTC

Field-specific reporting

Please select the one below that is the best fit for your research. If you are not sure, read the appropriate sections before making your selection.

☒ Life sciences ☐ Behavioural & social sciences ☐ Ecological, evolutionary & environmental sciences

For a reference copy of the document with all sections, see nature.com/documents/nr-reporting-summary-flat.pdf

Life sciences study design

All studies must disclose on these points even when the disclosure is negative.

Sample size	Cryo-EM sample size was not predetermined. Each cryo-EM dataset contained thousands of virus particles, from a single biological sample. The size of each dataset was based on instrument availability. Relevant statistics for image/particle numbers are provided under the cryo-EM section and in Fig. 7.
Data exclusions	Cryo-EM images of poor quality were removed based on visual inspection and assessment of the contrast transfer function.
Replication	Cryo-EM data collection was performed once for each sample. Fig 9c Individual data points represent mean value from two independent viral titrations.
Randomization	Cryo-EM data were split randomly into two half-datasets and independently refined. Fourier Shell Correlation between two half-datasets was used to provide a "gold-standard" estimate of the resolution of the cryo-EM maps.
Blinding	Blinding not relevant, analysis and collection of cryo-EM data did not require statistical interpretation and human bias is mitigated by established validation metrics.

Reporting for specific materials, systems and methods

We require information from authors about some types of materials, experimental systems and methods used in many studies. Here, indicate whether each material, system or method listed is relevant to your study. If you are not sure if a list item applies to your research, read the appropriate section before selecting a response.

Materials & experimental systems

n/a	Involved in the study	
☐	☒	Antibodies
☐	☒	Eukaryotic cell lines
☒	☐	Palaeontology and archaeology
☒	☐	Animals and other organisms
☒	☐	Human research participants
☒	☐	Clinical data
☒	☐	Dual use research of concern

Methods

n/a	Involved in the study	
☒	☐	ChIP-seq
☒	☐	Flow cytometry
☒	☐	MRI-based neuroimaging

Antibodies

Antibodies used	4G4 (hybridoma culture supernatant and recombinant expiCHO); E16 (recombinant expiCHO); Goat anti-mouse Immunoglobulins antibody, HRP conjugate (Dako, Cat: P0447); IRDye 800CW Goat anti-human IgG secondary (LiCor, Cat: 926-32232); AlexaFluor 488-conjugated goat anti-mouse IgG (H+L) secondary (Invitrogen; Cat: A-11001)
Validation	4G4 mab - Clark et al. J Gen Virol. 2007 Apr;88(Pt 4):1175-83.<br>Can be found here: https://eshop.uniquest.com.au/ns1-protein-monoclonal-antibody-mmabs-4g4/<br>E16 mAb - Nybakken et al. Nature. 2005. 437, 764-769, doi:10.1038/nature03956<br>Goat anti-mouse Immunoglobulins antibody, HRP conjugate - https://www.agilent.com/en/product/immunohistochemistry/antibodies-controls/secondary-antibodies/goat-anti-mouse-immunoglobulins-hrp-(affinity-isolated)-153239<br>IRDye 800CW Goat anti-human - https://www.licor.com/bio/reagents/irdye-800cw-goat-anti-human-igg-secondary-antibody<br>AlexaFluo 488-conjugated goat anti-mouse - https://www.thermofisher.com/antibody/product/Goat-anti-Mouse-IgG-H-L-Cross-Adsorbed-Secondary-Antibody-Polyclonal/A-11001

Eukaryotic cell lines

Policy information about cell lines

Cell line source(s)	C6/36 cells (Aedes albopictus) - sourced ATCC CRL1660<br>BSR cells (Mesocricetus auratus, baby hamster kidney) - sourced ATCC CCL-10<br>Vero cells (Cercopithecus aethiops, African green monkey kidney) - sourced ATCC CRL1586
Authentication	Cell lines were not genetically confirmed, but their morphologies were visually confirmed.
Mycoplasma contamination	Cell lines tested negative for mycoplasma.
Commonly misidentified lines<br>(See ICLAC register)	No commonly misidentified cell lines were used.
